# Huangkui capsule combined with finerenone attenuates diabetic nephropathy by regulating the JAK2/STAT3 signaling pathway based on network pharmacology, molecular docking, and experimental verification

**DOI:** 10.3389/fphar.2025.1625286

**Published:** 2025-08-04

**Authors:** Xuanke Liu, Chunjiang Zhang, YanJie Fu, JingJing Dai, Jiaying Lu, Gang Liu, Xiaoping Yang

**Affiliations:** Department of Nephrology, The First Affiliated Hospital of Shihezi University, Shihezi, China

**Keywords:** finerenone, diabetic nephropathy, JAK2/STAT3 signaling pathway, network pharmacology, molecular docking, kinetic simulation

## Abstract

**Introduction:**

Diabetic nephropathy (DN) is a serious complication of diabetes with limited therapeutic options. Although Huangkui capsule (HKC) and finerenone individually show potential in DN management, their combined mechanism remains unclear. This study aimed to explore the therapeutic effects and underlying mechanisms of HKC and finerenone combination for DN.

**Methods:**

An integrative approach combining network pharmacology, molecular docking, kinetic simulations, and experimental validation was employed in this study. Active components of HKC and finerenone, along with their potential targets, were identified through database mining. A “drug-component-target-disease” network was constructed, and interactions with the JAK2/STAT3 pathway were validated. *In vivo*, DN mice were treated with HKC, finerenone, and their combination (CDI group), while *in vitro*, HK-2 cells were treated with quercetin (a core HKC component) and finerenone. The binding index between quercetin and finerenone was analyzed by using the Chou–Talalay method.

**Results:**

Network pharmacology identified three core HKC components (quercetin, myricetin, and gossypetin) and 11 key targets (e.g., JAK2, STAT3, and AKT1). Molecular docking revealed strong binding affinity between quercetin–finerenone and JAK2/STAT3 (ΔG = −65.465 kcal/mol for STAT3-quercetin). In DN mice, combined therapy significantly reduced 24-h urinary protein (358.54 ± 21.21 mg/L vs. 1046.48 ± 72.84 mg/L in the model group, p < 0.001), improved serum creatinine/urea nitrogen, and downregulated IL-6/TNF-α expression. It also suppressed pro-apoptotic gene (*Bax, Caspase-3/8,* and *PARP*) activity while upregulating that of Bcl-2. Histopathology showed reduced tubular injury markers (NGAL and KIM-1) and fibrosis (p < 0.05). In HK-2 cells, quercetin + finerenone synergistically inhibited apoptosis and inflammation (p < 0.05), and the combined index (cl) was calculated to be less than 1. STAT3 overexpression exacerbated inflammation/apoptosis, which was reversed by combined treatment (p < 0.01).

**Conclusion:**

HKC combined with finerenone mitigates DN progression by inhibiting the JAK2/STAT3 pathway and reducing inflammation, apoptosis, and tubular injury. These findings provide a mechanistic basis for clinical application of this combination therapy.

## 1 Introduction

Diabetes mellitus (DM) is a metabolic disorder characterized by chronic hyperglycemia resulting from impaired insulin secretion or action. Uncontrolled DM frequently causes multi-organ damage, particularly affecting the ocular, renal, vascular, and nervous systems. Among these complications, diabetic nephropathy (DN) is a severe microvascular inflammatory manifestation and a leading cause of end-stage renal disease (ESRD) worldwide (Naaman and Bakris, 2023). Chronic low-grade inflammation and apoptosis play central roles in DN progression. Hyperglycemia activates the nuclear factor kappa B (NF-κB) and NLR family pyrin domain-containing 3 (NLRP3) inflammasome pathways, increasing pro-inflammatory cytokine (tumor necrosis factor alpha (TNF-α), interleukin-1β (IL-1β), and (IL-6) levels, and it damages glomerular endothelial cells, podocytes, and renal tubules (Islam et al., 2017). Additionally, hyperglycemia upregulates the Bax/Bcl-2 ratio and activates caspase-3, promoting tubular epithelial cell apoptosis and renal fibrosis. Notably, podocyte apoptosis disrupts the glomerular filtration barrier, which is a key contributor to DN-associated proteinuria (Mohandes et al., 2023).

The pathogenesis of DN has evolved from a glomerulocentric view to emphasize proximal tubular pathology. Although glomerular changes do contribute, they may not be the primary determinant of renal outcomes (Zeni et al., 2017; Gilbert, 2017). Up to 51% of diabetic patients exhibit renal impairment without detectable proteinuria (Tang et al., 2019), and ∼7% patients develop nonfunctional glomerular atrophy at the Bowman’s capsule–tubular junction (White et al., 2008). Even normoalbuminuric patients may show abnormal glomerulo-tubular junctions inversely correlated with creatinine clearance (White et al., 2008). Mechanistically, tubular injury can initiate podocyte damage via paracrine signaling (Hasegawa et al., 2013). Beyond their conventional roles, renal tubules critically mediate diabetes-induced inflammation and apoptosis. Tubular epithelial cell injury triggers a vicious cycle of podocyte dysfunction, glomerular damage, interstitial inflammation, and fibrosis, accelerating renal decline (Chevalier, 2016). Thus, targeting tubular lesions has become a key therapeutic strategy.

Huangkui capsule (HKC), a standardized preparation of traditional Chinese medicine (approval number: Z19990040) derived from *Abelmoschus manihot* (L.), contains bioactive flavonoids such as hypericin, quercetin, rutin, and myricetin. Clinical studies have demonstrated that HKC exhibits therapeutic effects in reducing inflammation and proteinuria and preserving renal function in patients with chronic glomerulonephritis, IgA nephropathy, and DN (Gu et al., 2021). Quercetin, a major bioactive component of HKC, inhibits tubular cell apoptosis via the phosphoinositide 3-kinase/AKT serine/threonine kinase (PI3K/AKT) signaling pathway (Liu et al., 2024). Finerenone (BAY94-8862), a novel non-steroidal mineralocorticoid receptor antagonist (MRA), has been shown to possess superior receptor selectivity and renal protective efficacy, as evidenced by the FIDELIO-DKD trial and subsequent investigations (Bakris et al., 2020; Yao et al., 2023a). Prior research on DN treatment predominantly focused on monotherapies, such as the use of HKC or finerenone alone. Although these approaches partially alleviated DN symptoms, their overall efficacy was limited, and adverse reactions were frequently observed. Consequently, there is an urgent need for innovative therapeutic strategies. In recent years, combination therapy has emerged as a promising direction for addressing untreated DN. Previous studies have indicated that combining HKC with glibenclamide (enhancing glucose metabolism) (Liu et al., 2012) or metformin (preventing fibrosis through Klotho/transforming growth factor beta 1 (TGF-β1)/p38 mitogen-activated protein kinase (p38MAPK) pathway blockade) (Gu et al., 2021) can significantly enhance therapeutic outcomes, thereby supporting the potential of combination therapies.

Network pharmacology has transformed traditional medicine research by systematically mapping disease–gene networks, compound–target interactions, and synergistic therapeutic mechanisms (Xu et al., 2020; Yan et al., 2024; Lu et al., 2022; Bi et al., 2024). This approach has proven valuable for deciphering the pharmacological basis of traditional Chinese medicine (TCM) in DN. For example, Wang et al. used network pharmacology and molecular docking to identify dehydromiltrone (DHT) from *Salvia miltiorrhiza* as a key bioactive compound that inhibits mesangial cell phenotypic switching via the PI3K/AKT pathway (Wang et al., 2023). Similarly, Yan et al. combined serum pharmacochemistry and network analysis to identify active components in *Alpiniae oxyphyllae* fructus (AOF), such as cubebin, which attenuates DN-associated cellular senescence by modulating signal transducer and activator of transcription 3 (STAT3) and PI3K/AKT signaling (Yao et al., 2023b). Further supporting this paradigm, Deng et al. integrated UPLC-MS/MS, network pharmacology, and transcriptomics to elucidate the multi-component synergy of *Schisandra chinensis* fruit mixture in DN, identifying schisandrin and quercetin as core bioactive compounds that ameliorate renal fibrosis through JAK2/STAT3 and TGF-β/Smad pathway inhibition (Deng et al., 2025). Expanding on these findings, our study employs an integrated multi-omics strategy—combining network pharmacology, molecular docking, dynamics simulations, and experimental validation—to investigate how HKC and finerenone synergistically alleviate tubular inflammation and apoptosis in early DN, with a focus on Janus kinase 2 (JAK2/STAT3) pathway inhibition.

## 2 Materials and methods

### 2.1 Materials

#### 2.1.1 Database and software database

The databases used were SwissTarget (www.ncbi.nlm.nih.gov/geo/), SuperPred (https://prediction.charite.de/index), GEO (https://www.ncbi.nlm.nih.gov/geo/), PubChem (https://pubchem.ncbi.nlm.nih.gov/), UniProt (https://www.uniprot.org/), DisGeNET (https://www.disgenet.org/), GeneCards (https://www.genecards.org/), OMIM (https://www.omim.org/), STRING (https://cn.String-db.org/), RCSB PDB (https://www.rcsb.org/), DAVID (https://davidbioinformatics.nih.gov/), and WeChat Health message (https://www.bioinformatics.com.cn/). The operating software applications were as follows: Cytoscape 3.9.1, AutoDockTools 1.5.7, Gromacs 2021.5, LigPlus + v2.2, and CompuSyn 3.0.1.

### 2.2 Animals

All mice were housed in the laboratory animal room of Shihezi University School of Medicine (temperature 22°C–26°C, body temperature 20°C–26°C, and humidity 50%–70%). The experiment was approved by the Ethics Committee of Shihezi University.

### 2.3 Drugs and reagents

Huangkui capsules [Z19990040, Jiangsu, China, specification 0.5 g *30 capsules *10 boxes]; finerenone (Chinese medicine license HJ20220057, Bayer, Germany, specification 10 mg/tablet); quercetin and finerenone (MCE, United States, product numbers HY-18085 and HY-111372).

## 3 Methods

### 3.1 Network pharmacology, molecular docking, and molecular dynamics simulations

#### 3.1.1 Target acquisition of HKC and finerenone

The active ingredients of HKC were initially identified through HPLC-Q-TOF-MS/MS analysis by Liu et al. (2013) and the comprehensive literature review of Li et al. (2021) yielding 50 candidate compounds. These compounds were subsequently screened using the TCMSP database with the selection criteria of oral bioavailability (OB) ≥10% and drug-likeness (DL) ≥0.1, resulting in 15 core components. These thresholds, which are well-established in pharmacophore analysis, effectively excluded approximately 85% of non-drug-like molecules while preserving flavonoids with demonstrated renal protective properties. SMILES expressions of the candidate compounds obtained from the PubChem database were used to search for relevant targets in SwissTarget and SuperPred databases, and finally, they were corrected with the UniProt database to remove duplicates.

#### 3.1.2 Screening of the targets of DN by HKC and finerenone

The disease targets of DN were searched in DisGeNET, GeneCards, and OMIM databases with “diabetic nephropathy” as the keyword, and the union of the three databases was taken as the disease target of DN. The targets of HKC and finerenone in the treatment of DN were obtained by the intersection of DN disease targets in a Venn diagram and the drug targets obtained in Section 2.1.1.

#### 3.1.3 Construction of the “drug-component-target-disease” network diagram

The network topology parameters of each node were calculated by Network Analyzer plug-in, and the core components of HKC and finerenone in the treatment of DN were screened according to the degree value. Higher degree values correlate with broader pharmacological effects.

#### 3.1.4 Protein interaction network and core target acquisition

The MCC algorithm in the CytoHubba plug-in was used to analyze the proteins in the PPI network to obtain the key targets. Finally, the intersection of the targets obtained by the two methods was considered the core target of HKC and finerenone in the treatment of DN (Ji et al., 2023).

#### 3.1.5 GO and KEGG enrichment analysis

The module with the highest score was selected to generate a new PPI network. The network nodes were adjusted according to the degree value to obtain the core protein module. The DAVID database platform was used to perform GO and KEGG enrichment analyses of the module targets, and finally, the micro-bioinformatics mapping and ClueGO plug-in were used to draw, respectively.

#### 3.1.6 GEO database analysis

The human diabetic nephropathy transcriptome chip GSE30122 was searched in the GEO database, and the renal tubular data samples were selected for analysis (including 10 cases of DN renal tubular samples and 24 cases of normal renal tubular samples). The key targets of the PPI network analysis were combined with the MCODE plug-in, and the samples were normalized using the normalize function. Genes with |logFC| ≥ 1.5 and adjusted p-value < 0.05 were screened, and finally, volcano map, heat map, and GSEA enrichment analysis map were drawn by micro-bioinformatics.

#### 3.1.7 Molecular docking analysis

The optimal docking conformation was screened according to the principle of “the lower the binding energy between the ligand molecule and the receptor protein, the more stable the molecule is.” The PyMol software application was used for 3D visualization mapping, the PDB format file of the docking complex was the output, and the 2D structure diagram was drawn by LigPlus.

### 3.2 Animal experiment validation

#### 3.2.1 Modeling, grouping, and drug administration

After a 12-h fast, the model group received an intraperitoneal injection of streptozotocin (STZ) (100 mg/kg, sourced from Beijing, product number S8050), while the normal group was administered an equivalent volume of citric acid–sodium citrate buffer. Post-modeling, mice resumed feeding, and fasting blood glucose levels were measured on days 3, 7, and 10. The DM model was deemed successful if blood glucose levels were ≥16.7 mmol/L on three consecutive measurements. Subsequently, 24-h urinary protein levels were monitored, and a DN model was confirmed when 24-h urinary protein exceeded the concentration of 30 mg (Jiang et al., 2024). During the modeling process, eight mice that failed to develop the condition or died were excluded, leaving 40 mice that met the DN criteria. These were randomly divided into four groups: the model group, the HKC group (0.45 g/kg/d), the finerenone group (1.55 mg/kg/d), and the combined drug group, with 10 mice in each group. Each treatment group received the corresponding dose via oral gavage, while the control and model groups were given an equivalent volume of saline, administered once daily for 8 weeks. The body weight of mice was measured at 0, 2, 4, 6, and 8 weeks. After the end of drug intervention, the mice were sacrificed by eyeball blood sampling, serum was separated, and the bilateral kidneys of mice were weighed. The right kidney was stored in the refrigerator at −80°C, and the left kidney was soaked in 4% paraformaldehyde for paraffin sections.

#### 3.2.2 Urine protein and renal function tests were performed

At 0, 2, 4, 6, and 8 weeks after administration, the mice in each group were placed in metabolic cages for 24 h to collect urine samples. Serum samples were collected from the mice, and the total urinary protein, serum creatinine, and serum urea nitrogen were measured using the CBB method and enzymatic kits (Jiancheng Biological, Nanjing, China).

#### 3.2.3 Detection of inflammatory markers

Serum samples of mice were collected, and the levels of IL-6 and TNF-α in each group were quantified by mouse IL-6 and a TNF-α ELISA kit (Jianglai Bio, Shanghai, China).

#### 3.2.4 Renal histopathology and IHC analysis

The left kidney tissue was fixed with paraformaldehyde, dehydrated, embedded, and underwent other steps to form paraffin blocks. The paraffin tissue with a thickness of 3 μm was cut to prepare sections, and they were finally subjected to HE, Masson, and PAS pathological staining (Huang et al., 2020; Xiu et al., 2021). The pathological changes of the glomeruli and renal tubulointerstitium were observed under the microscope. Finally, the number of nuclei in the glomeruli and the stained area of the glycogen and collagen fibers were analyzed and quantified by ImageJ software. For IHC staining, sections were stained with antibodies to neutrophil gelatinase-associated lipocalin (NGAL) (1:200) (BosterBio, Wuhan, China, No. 83221-2-RR) and kidney injury molecule-1 (KIM-1) (1:300) (Proteintech, Wuhan, China, 26991-1-AP) and photographed with an inverted fluorescence microscope (Nikon, Japan). The positive areas were analyzed using ImageJ software to compare the improvement in renal tubular damage in each group.

#### 3.2.5 IF was co-stained with TUNEL

The left kidney fixed with paraformaldehyde was dehydrated and embedded to form paraffin blocks. Paraffin sections with a thickness of 3 μm were prepared through microtomy. The sections were permeabilized with 0.3% Triton X-100, then blocked with 5% goat serum at 37 °C, and primary antibodies MCP-1 (1:200) and MIP-2 (1: 200) (Proteintech, Wuhan, China, 26161-1-AP, 26791-1-AP) were incubated at 4 °C overnight and then incubated with CoraLite594-conjugated goat anti-rabbit IgG (H + L) (Proteintech, Wuhan, China) at 37 °C in the dark the next day, according to the instructions for the TUNEL fluorescence assay kit (Vazyme, Nanjing, China). The sections were stained with TUNEL and labeled with nucleic acid fragments of apoptotic cells. Finally, the nuclei were counterstained with DAPI in the dark, and the slides were sealed and photographed with an inverted fluorescence microscope (Nikon, Japan). ImageJ software was used to analyze the relative fluorescence quantitative values and compare the local inflammation and apoptosis of the renal tubules in each group.

#### 3.2.6 Real-time fluorescent quantitative PCR

Total RNA was extracted from the mice kidney using the RNA extraction kit (OMEGA, Switzerland), and cDNA was reverse-transcribed from total RNA using the RevertAid RT reverse transcription kit (Thermo Fisher Scientific, United States). Then, qRT-PCR analysis was performed on a SLAN-96S real-time PCR system using SYBR Green reagent (Vazyme, Nanjing, China). The analyzed genes and primer sequences are shown in Supplementary Table S6, along with all the front and reverse primers (Sangon, Shanghai, China). Renal tissue mRNA levels were normalized to the endogenous housekeeping gene β-actin and relative to calibrator samples using the 2^−ΔΔCT^ method. Mouse kidneys from the control group were used as calibration samples for *in vivo* analysis.

#### 3.2.7 Western blot

Renal tissues (30 mg) were homogenized in RIPA lysis buffer and subsequently centrifuged at 12,000 × g for 30 min at 4 °C. Protein concentrations were quantified using the BCA assay (Pierce™, Thermo Fisher Scientific, United States), with bovine serum albumin serving as the standard. Equal amounts of protein were resolved by SDS-PAGE and transferred onto PVDF membranes. The membranes were blocked with 5% non-fat milk in TBST for 1 h at room temperature and then incubated overnight at 4 °C with the following primary antibodies: p-JAK2, p-STAT3 (Abcam, United Kingdom; catalog numbers ab108596 and ab267373), JAK2, STAT3, and caspase-3/cleaved caspase-3 (Bausch, Wuhan, China). Additionally, the protein expression levels of BAX, BCL-2, caspase-8/cleaved caspase-8 (p18), and PARP/cleaved PARP (Wanlei Biological, Shenyang, China) were detected. For loading control and normalization, all blots were simultaneously probed with a β-actin antibody (catalog number #4970, ZSGB-Bio, Beijing, China) as an internal reference. After incubation with HRP-conjugated secondary antibodies (ZSGB-Bio, Beijing, China) for 1 h at room temperature, protein bands were visualized using enhanced chemiluminescence (ECL). Quantitative analysis was performed using densitometric analysis via ImageJ software, with target protein expression normalized to β-actin within the same sample. Three independent biological replicates were analyzed under each condition.

### 3.3 Cell experiment verification

#### 3.3.1 Cell culture and experimental grouping

HK-2 cells were induced with LPS (10 μg/mL) (MCE, United States) combined with high glucose (30 mmol/L) to establish inflammation and apoptosis models (Xu et al., 2024) and then treated with quercetin (Que) and finerenone (Fine) (MCE, United States, drugs were dissolved in DMSO, concentration 0.1%). The experiment consisted of six groups: (1) control group; (2) high glucose (30 mmol/L); (3) LPS (10 μg/mL) + high glucose group; (4) LPS + high glucose + Que group; (5) LPS + high glucose + Fine group; (6) LPS + high glucose + CDI group; the cells were starved with serum-free medium for 12 h before LPS, Que, and Fine treatment. Then, the intervention time of different drugs was 24 h.

#### 3.3.2 Determination of drug toxicity and combination index

Drug toxicity was determined using the CCK-8 assay. HK-2 cells (1 × 10^4^ cells/well) was seeded into 96-well culture plates, and then the cells were treated with Que (0∼100 μM) and Fine (0∼100 μM) in six multiple-well plates per group at 24 and 48 h. The absorbance was detected at 450 nm, and the survival rate and inhibition rate were calculated as follows:

$$\text{Cell survival rate}=\left[\left(\text{experimental well}-\text{blank}\right)/\left(\text{control well}-\text{blank}\right)\right]\times 100\%.$$

$$\text{Cell inhibition rate}=\left[\left(\text{control well}-\text{experimental well}\right)/\left(\text{control well}-\text{blank well}\right)\right]\times 100\%.$$

According to the inhibition rate of the two compounds on the cells, Que and Fine were combined in a ratio of 1:2, and then the semi-inhibition concentration curve was calculated using the calculation software developed by Chou and Martin (Banerjee et al., 2021).

#### 3.3.3 Immunofluorescence

HK-2 cells (5 × 10^4^) were seeded in a six-well microplate with a slide (18 × 18 mm). After cell adhesion was stabilized, different drugs were added to the cells. HK-2 cells were fixed in 4% paraformaldehyde, permeabilized with 0.3% Triton X-100, washed, blocked with 5% goat serum at room temperature, and then incubated with antibodies monocyte chemoattractant protein-1 (MCP-1) (1:200) and macrophage inflammatory protein-2 (MIP-2) (1:200) at 4 °C overnight. The next day, the cells were incubated with fluorescein (FITC)-conjugated donkey anti-rabbit IgG (H + L) (Proteintech, Wuhan, China) at 37 °C in the dark. After washing, the cells were counterstained with DAPI, and the slides were photographed with an inverted fluorescence microscope.

#### 3.3.4 Detection of apoptosis

Each group of HK-2 cells was treated with specific drugs, followed by digestion using 0.25% trypsin without EDTA. Since the transfected cells showed green fluorescence, each group of cells was treated with an Annexin-V FITC/PI kit (Elabscience, Wuhan, China) according to the instructions, and then reagents were added and incubated in the dark for 15 min. Apoptotic cells were determined by flow cytometry (EASY CELL, Shenzhen, China). Finally, FlowJo software (version 10.6.2) was used to analyze the percentage of apoptotic cells.

#### 3.3.5 Western blot

HK-2 cells were treated with different drugs in each group, the cells were collected, and the following steps were used to extract kidney tissue protein. All blots were simultaneously probed with a β-actin antibody as an internal reference. After incubation with HRP-conjugated secondary antibodies for 1 h at room temperature, protein bands were visualized using ECL. Quantitative analysis was performed using densitometric analysis via ImageJ software, with the target protein expression normalized to β-actin within the same sample. Three independent biological replicates were analyzed under each condition.

#### 3.3.6 Lentiviral transfection design and grouping

Based on the screening of network pharmacology, molecular docking, and kinetic simulation, STAT3 was selected as the core target, and the STAT3 overexpression lentiviral vector (Jima Gene, Suzhou, China) was constructed. HK-2 cells were transfected according to the instructions, and different MOI values (10–100) were set. The titer with high infection efficiency (MOI = 60) that did not affect cell activity was selected for subsequent experiments. After transfection, puromycin (5 μg/mL) was used to screen the stable strain. Finally, the experimental animals was divided into eight groups: (1) LV-NC group; (2) LV-NC + high glucose group; (3) LV-NC + high glucose + LPS group; (4) LV-NC + high glucose + LPS + CDI group; (5) LV-STAT3 group; (6) LV-STAT3+ high glucose group; (7) LV-STAT3+ high glucose + LPS group, and (8) LV-STAT3+ high glucose + LPS + CDI group. The subsequent experiments were carried out according to the grouping.

#### 3.3.7 Apoptosis after overexpression of STAT3

For the stable HK-2 cell lines containing LV-STAT3 and LV-NC, different drugs were added to the cells, and the following steps were the same as before. Since the transfected cells showed green fluorescence, each group of cells was treated with an Annexin-V APC/7AAD kit (Elabscience, Wuhan, China) according to the instructions, and the subsequent steps were the same as above for the general cell loss and apoptosis assay. Finally, FlowJo software was used to analyze the percentage of apoptosis and compare the changes of apoptosis after overexpression of STAT3 in each group.

#### 3.3.8 Western blot after overexpression of STAT3

For the stable HK-2 cell line containing LV-STAT3 and LV-NC, the cells were collected, and the kidney tissue protein was extracted. Subsequent methods were performed as described above for Western blot experiments. The primary antibodies that were needed to be tested were as follows: P-STAT3, IL6, TNF-α, NGAL, and KIM-1. For loading control and normalization, all blots were simultaneously probed with a β-actin antibody as an internal reference. After incubation with HRP-conjugated secondary antibodies for 1 h at room temperature, protein bands were visualized using ECL. Quantitative analysis was performed using densitometric analysis via ImageJ software, with target protein expression normalized to β-actin within the same sample. Three independent biological replicates were analyzed under each condition.

## 4 Statistical analysis

Data are presented as mean ± standard deviation (‾X ± SD), as indicated. Normality was assessed using the Shapiro–Wilk test. Multiple-group comparisons were analyzed by one-way ANOVA. For time-course experiments, two-way ANOVA with post-test was used. Statistical significance was set at p-value: *< 0.05, **< 0.01, and ***< 0.001. All analyses were performed using SPSS 27.0 software, and GraphPad Prism 9.0 software was used for drawing.

### 4.1 Network pharmacology, molecular docking, and molecular dynamics simulations

#### 4.1.1 Target acquisition of HKC and finerenone

A total of 15 HKC active ingredients were screened by the TCMSP database, and one active ingredient was obtained by finerenone target through the DrugBank database.

#### 4.1.2 Screening of the targets of DN by HKC and finerenone

**FIGURE 1:**
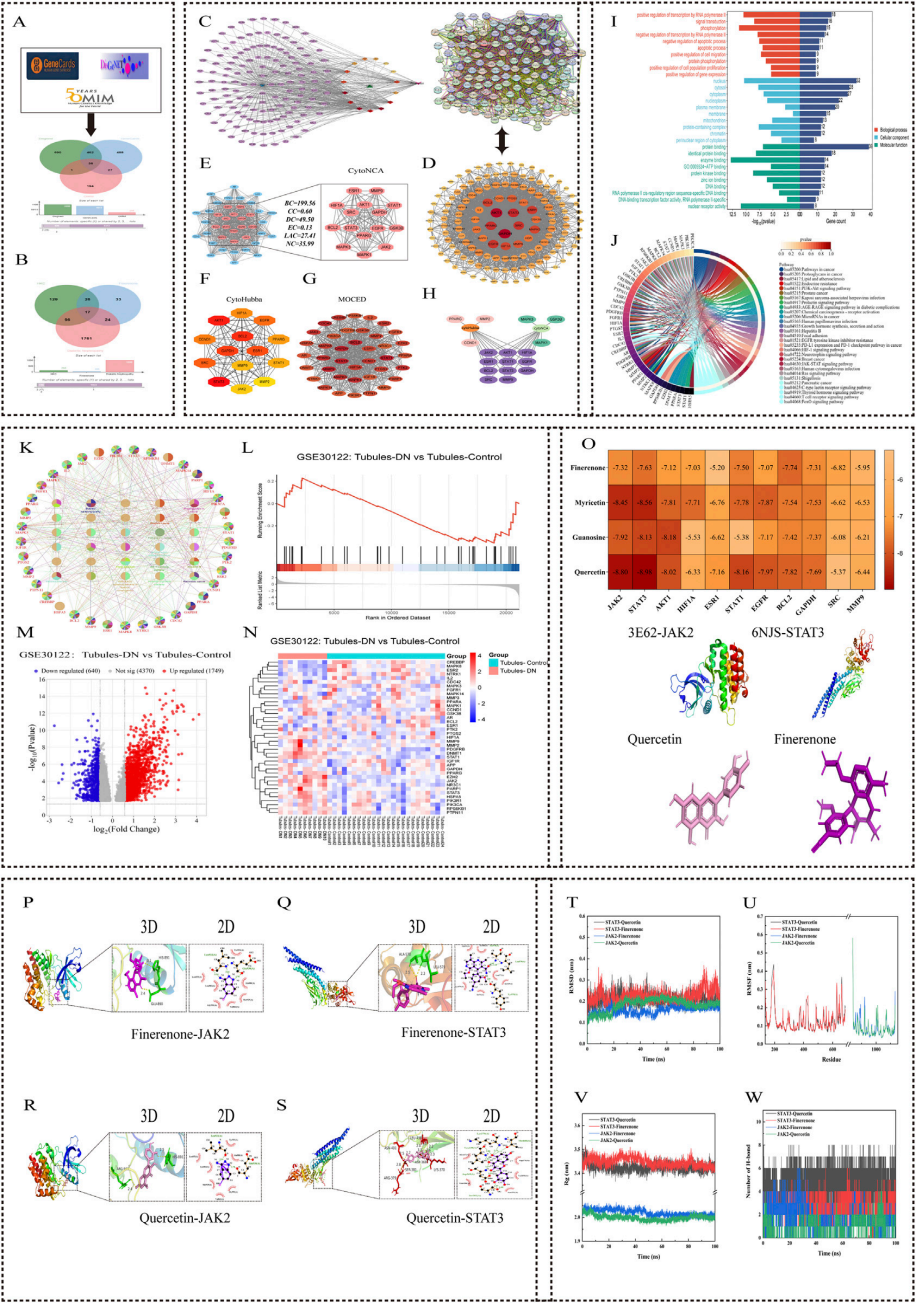
Network pharmacology and molecular docking analysis of HKC and Finerenone in DN treatment. **(A, B)** Venndiagrams of drug-disease overlapping targets. HKC and Finerenone shared 137 common targets with DN. **(C)** Drug-component-target-disease network. Top three HKC component Degrees: Quercetin (101), Myricetin (101), Gossypetin (101). **(D, E)** PPI network and core targets. 11 key targets (JAK2, STAT3, AKT1, etc.) identified via CytoHubba. **(F)** Subnetwork of top-ranked targets from MCC algorithm analysis, highlighting high-confidence interactions. **(G)** Clustering analysis of PPI network using MCODE, showing densely connected target modules. **(H)** Heatmap of topological parameter scores (BC, CC, DC) for core targets. **(I, J)** GO/KEGG enrichment. JAK/STAT pathway was the most enriched. **(K)** Bubble plot of KEGG pathways ranked by significance (-log10 (p-value)). **(L–N)** GSE30122 analysis. JAK2/STAT3 expression elevated in DN renal tubules (logFC≥1.5, p<0.05). **(O)** Molecular docking binding energy. STAT3-Quercetin complex showed strongest binding affinity. **(P–S)** 3D and 2D interaction diagram of quercetin-JAK2/STAT3 and finerenone-JAK2/STAT3 binding pocket with hydrogen bond formation. **(T)** Root Mean Square Deviation (RMSD) trajectory of molecular dynamics simulations for protein-ligand complexes. **(U)** Root Mean Square Fluctuation (RMSF) analysis of amino acid residues in simulated complexes. **(V)** Radius of gyration (Rg) plot demonstrating structural stability during simulations. **(W)** Hydrogen bond occupancy analysis between core components and target proteins over 100ns simulation.

The targets of HKC and finerenone on DN were obtained from the DisGeNET, GeneCards, and OMIM databases, respectively, and 1,189, 1,013, and 258 DN-related targets were obtained. Finally, these disease targets were intersected with 270 drug targets. A total of 137 intersection targets were obtained as the targets of HKC and finerenone on DN (Figures 1A,B).

#### 4.1.3 Construction of the “drug-component-target-disease” network diagram

The 137 intersection targets obtained were input into Cytoscape software for network diagram construction. Topologic analysis of each node in the network obtained 2,106 nodes and 2,691 edges, and the degree value of nodes was used to determine their importance (Supplementary Table S4). The top three compounds in HKC were quercetin, myricetin, and gossypetin. These results indicate that these components can act on most of the targets in DN and may be important effectors of HKC and finerenone in the treatment of DN (Figure 1C).

#### 4.1.4 The protein interaction network and acquisition of core targets

The median values of BC, CC, DC, EC, LAC, and NC of each network node were calculated by CytoNAC plug-in. After three calculations, 14 targets were obtained after screening the median values of the above topological parameters of each node in the sub-network, and the sub-network diagram was drawn. After analyzing the PPI network with MCC algorithm in the CytoHubba plug-in, 14 targets with the highest prediction scores were obtained, and a new sub-network diagram was drawn. Twenty-eight targets were obtained after the integration of the targets obtained by the two methods. Among them, JAK2, STAT3, AKT1, hypoxia-inducible factor 1 subunit alpha (HIF1A), estrogen receptor 1 (ESR1), STAT1, epidermal growth factor receptor (EGFR), B-cell lymphoma 2 (BCL2), glyceraldehyde-3-phosphate dehydrogenase (GAPDH), SRC proto-oncogene, non-receptor tyrosine kinase (SRC), and matrix metallopeptidase 9 (MMP9) were identified in both plug-ins. These 11 targets may be important regulatory sites for the treatment of DN by HKC and finerenone (Figures 1D,E).

#### 4.1.5 GO and KEGG enrichment analysis

A protein module with a comprehensive score of 28.421 was obtained after analyzing the PPI network by MCODE plug-in. This module was composed of 39 protein targets and was the core site in multiple regulatory mechanisms of the PPI network. The GO enrichment results of this protein cluster included 299 biological processes (BPs), 37 cellular components (CCs), and 77 molecular functions (MFs). Among them, the module targets were mainly enriched in cell components such as the cytoplasm, mitochondria, and nucleus, which were mainly involved in cell signal transduction, apoptosis, and protein phosphorylation. Involving molecular functions such as protein binding, ATP binding, and enzyme binding, the top-10 most significantly enriched items in each group were selected to draw the horizontal bar chart. The KEGG enrichment results included 141 signaling pathways. The string diagram of the top 30 pathways was drawn by the microbiotics and ClueGO plug-ins. These pathways were mainly related to cancer, endocrine metabolism, inflammatory response, and cell proliferation, among which, the JAK/STAT signaling pathway had the most enriched targets. These results indicated that HKC and finerenone played an important role in the regulation of DN (Figures 1I,J).

#### 4.1.6 GEO database analysis

The human diabetic nephropathy transcriptome chip GSE30122 (including 10 DN renal tubular samples and 24 normal renal tubular samples) was selected. The samples were normalized by the normalize function, and the genes with |logFC| ≥ 1 and adjusted p-value <0.05 were screened. Finally, 39 protein core targets were analyzed in the GSE30122 chip by the MCODE plug-in. The volcano map, heat map, and GSEA enrichment analysis map were used to draw the micro bioinformatics. It could be seen that JAK2 and STAT3 core proteins were upregulated in the renal tubules of DN patients than in normal renal tubules. This indicates that DN renal tubules activate the JAK2/STAT3 pathway (Figures 1L–N).

#### 4.1.7 Molecular docking analysis

The three core components obtained were verified by molecular docking with 11 core targets. As shown in Figure 1, the binding free energy of all complexes is less than -5 kcal/mol. The top components of HKC and finerenone with JAK2/STAT3 binding free energy were shown by 3D mapping, and they could form a relatively stable conformation by intermolecular forces such as hydrogen bonding, indicating that the three core components of HKC could have considerable binding relationships with the 11 core target proteins. Moreover, the three core components of HKC (quercetin, myricetin, and gossypetin) and finerenone on JAK2/STAT3 are the key to its regulatory mechanism (Figures 1O–S).

#### 4.1.8 Molecular dynamics simulation

A smaller root mean square deviation (RMSD) value indicated a better stability of the simulated system. After 100 ns of simulation, the graphical curves of the four small molecular–protein complexes fluctuated within the range of 0.2–0.3 nm, indicating that the overall simulated system had a small range of motion and good stability. The root mean square fluctuation (RMSF) reflects the flexibility and free movement of amino acid residues in the whole simulation process. The larger the RMSF fluctuation, the poorer the binding stability of small molecules to proteins. The effects of quercetin and finerenone on the degree of freedom of the residues in the same protein were similar. At the same time, the RMSF value of the key amino acid residues in the binding between the receptor protein and the small-molecule ligand was small, which was basically in the trough, indicating that these key amino acid residues had a small fluctuation range and maintained a stable conformation in the complex system. In addition, there are still some amino acid residues with a large fluctuation range, but these amino acid residues with a large fluctuation range are located in the loop region of the protein. Since the small-molecule ligand does not have this region to bind to the receptor protein, these amino acid residues are more flexible. In general, the RMSF fluctuation range of the four complex systems in the simulation was low, and the complex conformation was stable. The value of the radius of gyration (Rg) reflects the stability of the complex system. The larger the fluctuation of the Rg value, the more unstable the complex system is. The fluctuation range of Rg values of the four complexes does not exceed 0.2 nm, indicating that the complex system can maintain a stable and compact state during the simulation. The change in the number of hydrogen bonds was used to assess the magnitude of the force maintaining a stable binding between the two. The number of hydrogen bonds of the four complexes could be maintained at more than 3, which was consistent with the hydrogen bonds formed in the molecular docking process, and it indirectly verified the reliability of hydrogen bond binding in the molecular docking process. The binding free energy data of the motion trajectories obtained by molecular dynamics were calculated using MM/GBSA. The binding free energy is usually composed of two components, namely, the molecular mechanical term and the solvation energy, which reflects the affinity between the small-molecule drug and the protein target. The lower the binding free energy, the better the drug affinity and the better the drug efficacy. As shown in Supplementary Table S2, ΔGBind represents the binding free energy. The binding free energy level of the four complexes is maintained below −10, among which the STAT3–quercetin complex has the highest binding free energy of −65.465, indicating that these small molecules have a strong affinity with their corresponding proteins. The results were consistent with those of the above experimental analysis (Figures 1T–W).

### 4.2 Animal experiment verification

#### 4.2.1 DN model validation and urine protein and renal function tests

**FIGURE 2:**
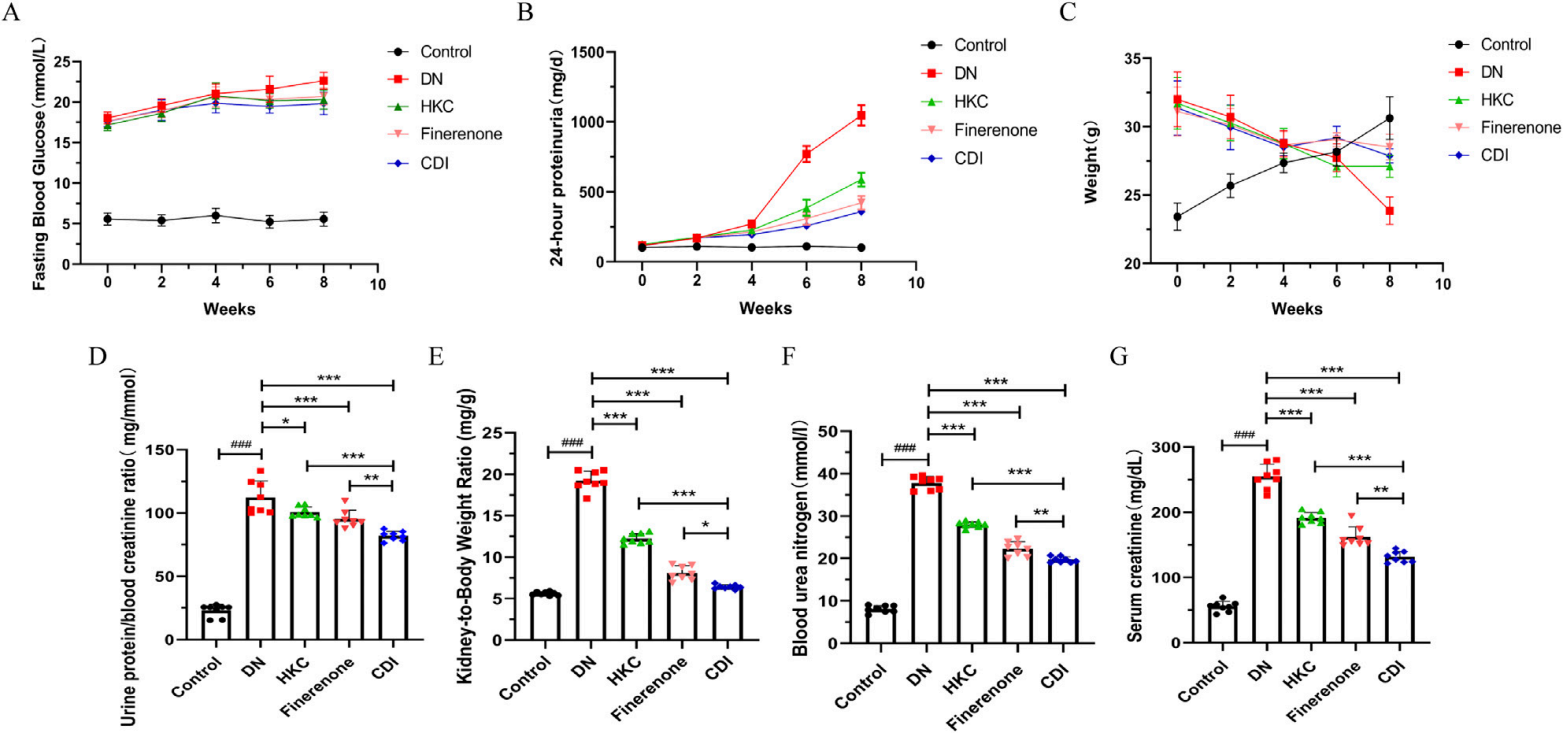
Effect of combination therapy on the identification of DN mouse model establishment and urinary protein levels (n = 8 per group, ‾X ± SD), which was analyzed by two-way ANOVA. **(A)** Time course of 24-h urinary protein excretion. Compared with the model group, proteinuria was significantly reduced in the CDI group (**p < 0.01). Monotherapy also demonstrated a significant reduction (*p < 0.01). **(B)** Time course of fasting blood glucose levels. Following STZ injection, fasting blood glucose levels in mice were consistently maintained above 16.7 mmol/L compared to that in the control group, confirming the successful establishment of the DM model. **(C)** Time course of mouse body weight. Mice injected with STZ exhibited a gradual decrease in body weight over time compared to the control group, further validating the establishment of the DM model. The effect of combination therapy on renal function in DN mice (n = 6, ‾X ± SD), which was analyzed by one-way ANOVA. **(D–F)** Serum creatinine, blood urea nitrogen, and urinary protein-to-serum creatinine ratio. The CDI group showed significant improvements compared to the model group (**p < 0.01) and the monotherapy group (*p < 0.05). **(G)** Renal hypertrophy index (kidney weight/body weight ratio). The CDI group exhibited a marked improvement compared to the model group (**p < 0.01) and the monotherapy group (*p < 0.05).

As depicted in Figure 2A, the 24-h urinary protein levels remained stable and exhibited no significant differences between groups during the first 2 weeks following STZ injection (p > 0.05). At 6 and 8 weeks post-injection, the urinary protein levels in the model group were significantly elevated than that in the control group (p < 0.01). Notably, the 24-h urinary protein levels in the CDI group were markedly lower than those in the monotherapy group (p < 0.001), as detailed in Supplementary Table S3. As illustrated in Figures 2B, C, the fasting blood glucose levels of mice injected with STZ were significantly higher than those of the control group over time and consistently exceeded 16.7 mmol/L. Concurrently, body weight progressively decreased relative to the control mice, confirming the successful establishment of the diabetic mouse model (Supplementary Tables S4,S5). As shown in Figures 2D–F, serum creatinine, urea nitrogen, and the creatinine/urea nitrogen ratio in the model group were significantly higher than those in the control group (p < 0.001). Furthermore, on comparing the HKC, finerenone, and CDI groups, the CDI group demonstrated significantly reduced serum creatinine, urea nitrogen, and urine protein/creatinine ratios (p < 0.01). Additionally, Figure 2G presents a comparison of the renal hypertrophy index (weight of both kidneys/body weight) across groups. The renal hypertrophy index was significantly higher in the model group than in the control group (p < 0.001) and significantly lower in the CDI group than in the monotherapy group (p < 0.01).

#### 4.2.2 Detection of inflammatory indicators

**FIGURE 3:**
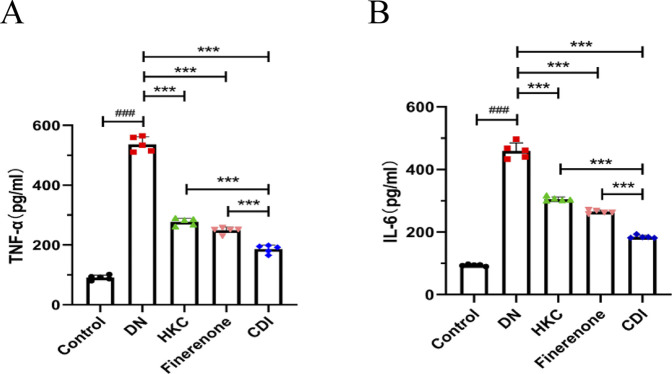
Anti-inflammatory effects of combined therapy in DN mice (n = 5 per group, ‾X ± SD): **(A,B)** analyzed by one-way ANOVA. Serum IL-6 and TNF-α levels. CDI group reduced IL-6 and TNF-α vs. model (***p < 0.001) and vs. monotherapy (***p < 0.001) groups.

Serum samples were collected from mice in each group, and inflammatory markers were measured using mouse IL-6 and TNF-α ELISA kits. As shown in Figures 3A,B, the results revealed that inflammatory markers in the model group were significantly elevated than in the control group (p < 0.001). In contrast, the HKC, finerenone, and CDI groups showed a notable reduction in inflammatory markers following drug intervention (p < 0.001). Furthermore, the CDI group exhibited a more pronounced decrease in inflammatory markers than the single-drug groups (p < 0.001).

#### 4.2.3 Real-time fluorescence quantitative PCR

**FIGURE 4:**
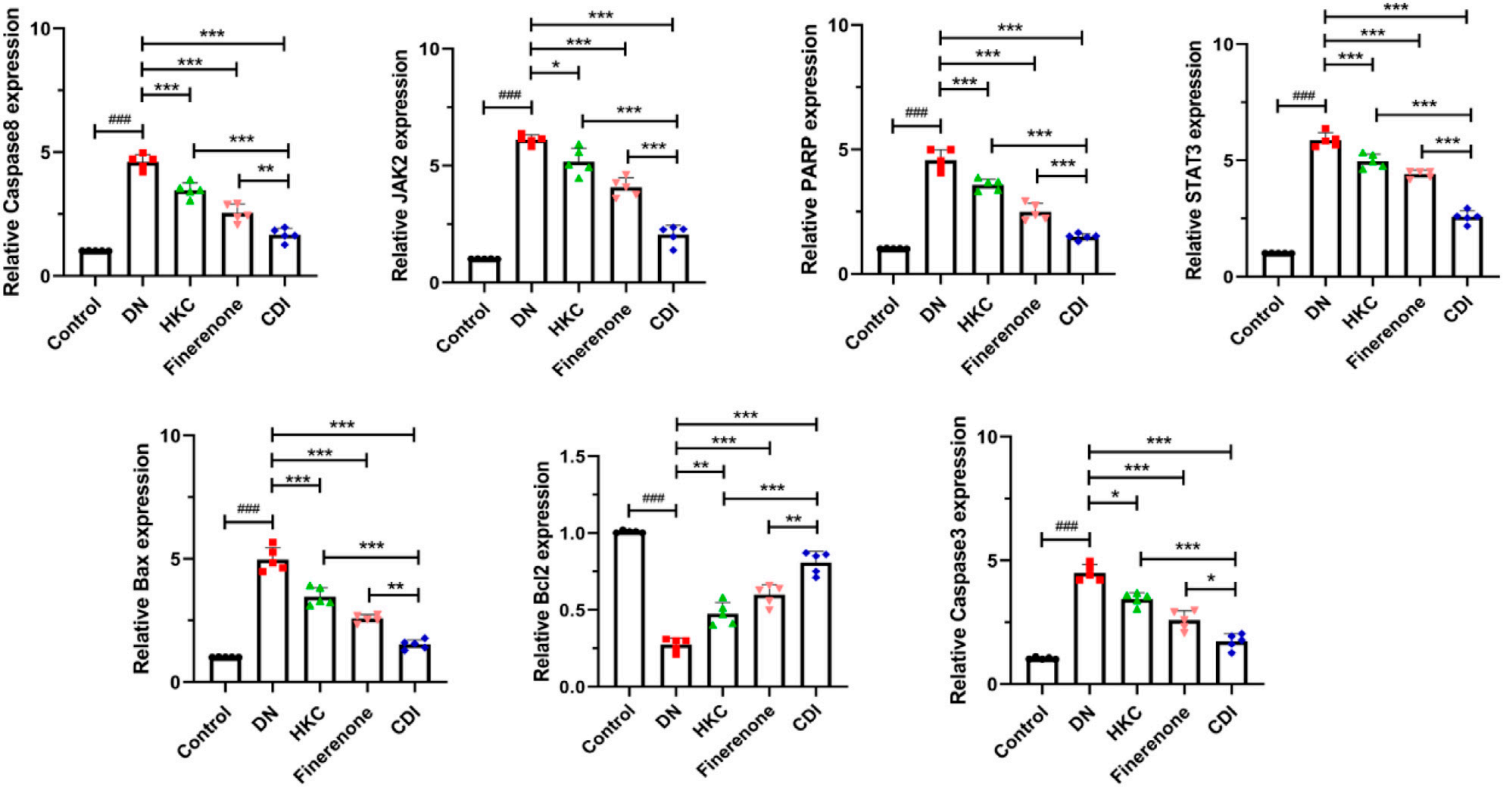
Regulation of apoptosis and signaling pathway-related genes in the renal tissue of DN mice by combined treatment (n = 5 per group, ‾X ± SD), which was analyzed by one-way ANOVA. A-G:RT-PCR analysis. CDI group showed downregulated *JAK2*, *STAT3*, *Bax*, *caspase-3/8*, and *PARP* expressions and upregulated *Bcl-2* vs. model (***p < 0.001) and vs. monotherapy (*p < 0.05) groups.

As shown in Figures 4A–G, the model group exhibited significantly elevated expression levels of *JAK2*, *STAT3*, *Bax*, *caspase-3*, *caspase-8*, and *poly (ADP-Ribose) polymerase(PARP)* genes than the control group (p < 0.001), while the expression of the *Bcl-2* gene was notably reduced (p < 0.001). In contrast, the HKC, finerenone, and CDI groups showed significantly lower expression levels of *JAK2*, *STAT3*, *Bax*, *caspase-3*, *caspase-8*, and *PARP* than the model group (p < 0.05). Notably, the CDI group demonstrated even lower expression levels of these genes than the single-drug groups (p < 0.05). Additionally, the expression of the *Bcl-2* gene was higher in the HKC, finerenone, and CDI groups than in the model group (p < 0.01), with the CDI group showing the highest *Bcl-2* expression among all treatment groups (p < 0.05). Finally, the before and after primer details for each gene can be found in Supplementary Table S4.

#### 4.2.4 Renal histopathology and IHC analysis

**FIGURE 5:**
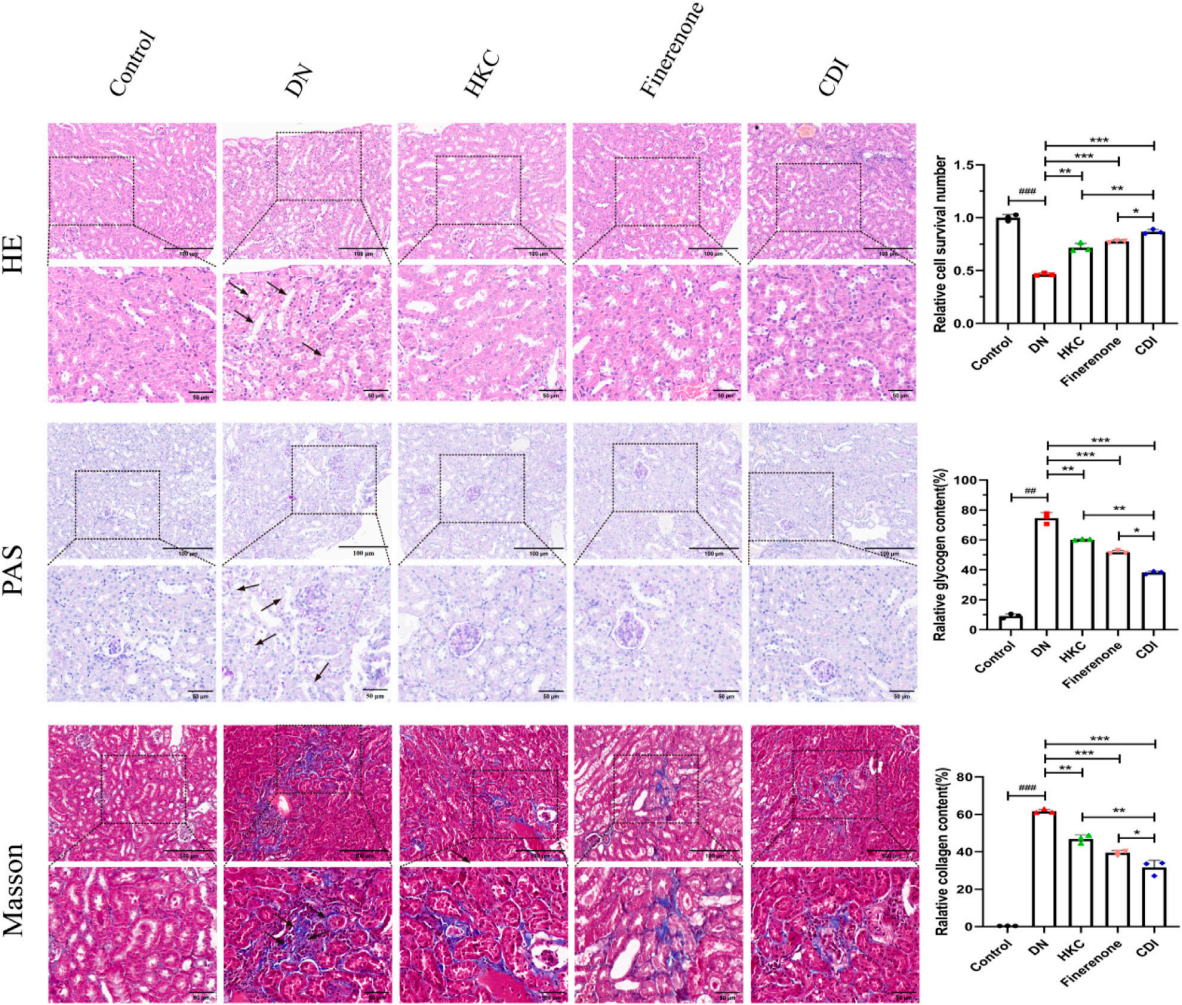
Histopathological improvement in renal tissue (n = 3 per group, scale bar 50/100 μm, ‾X ± SD), which was analyzed by one-way ANOVA. HE/PAS/Masson staining: the CDI group showed reduced tubular vacuolization, collagen deposition, and glomerular hypertrophy vs. model (***p < 0.001) and vs. monotherapy (*p < 0.05) groups.

**FIGURE 6:**
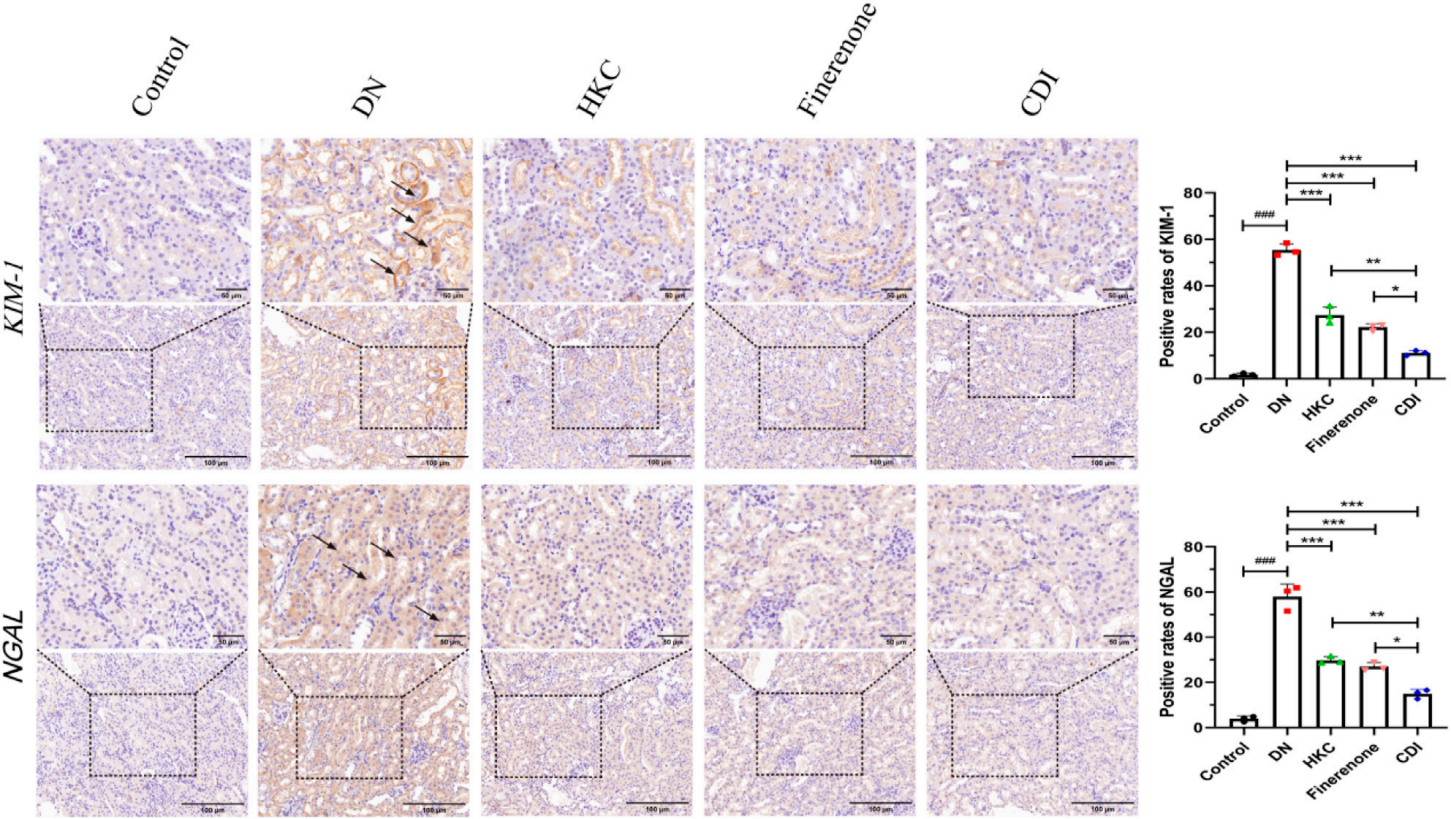
IHC analysis of tubular injury markers (n = 3 per group, scale bar 50/100 μm, ‾X ± SD), which was analyzed by one-way ANOVA. KIM-1/NGAL expression: the CDI group showed decreased KIM-1 (45%) and NGAL (52%) vs. model (***p < 0.001) and vs. monotherapy (*p < 0.05) groups.

The structure of the glomeruli and tubules in the control group was normal, and there was no collagen fiber and glycogen deposition. However, renal tubular epithelial vacuolization, nuclear exfoliation, uterine cavity hypertrophy, and interstitial inflammatory cell infiltration were observed in the model group. In addition, extensive collagen fibers and glycogen deposition were observed in glomeruli and renal interstitium, as shown in Figure 5 (HE, PAS, and Masson staining). Glomerular mesangial proliferation and hypertrophy could also be seen. All the above pathological changes were improved after the intervention of HKC, finerenone, and CDI, and the improvement in the CDI group was more significant than that in the monotherapy group. As depicted in Figure 5, the model group exhibited an increase in the number of glomerular nuclei, heightened glycogen deposition, and altered renal tubulointerstitial fibrosis compared to the normal group (p < 0.001). In contrast, the HKC, finerenone, and CDI groups showed a reduction in glomerular nuclei count, glycogen deposition, and renal tubular fibrosis (p < 0.01). Notably, the CDI group demonstrated less severe histopathological damage than the single-drug groups (p < 0.05). As illustrated in Figures 6I,H,C, immunohistochemical analysis revealed elevated expressions of KIM-1 and NGAL, which are markers of renal tubular injury, in the model group than in the control group (p < 0.001). However, drug intervention in the HKC, finerenone, and CDI groups led to improved renal tubular injury (p < 0.01). Among these, the CDI group showed greater improvement in renal tubular injury than the single-drug groups (p < 0.05).

#### 4.2.5 IF was co-stained with TUNEL

**FIGURE 7:**
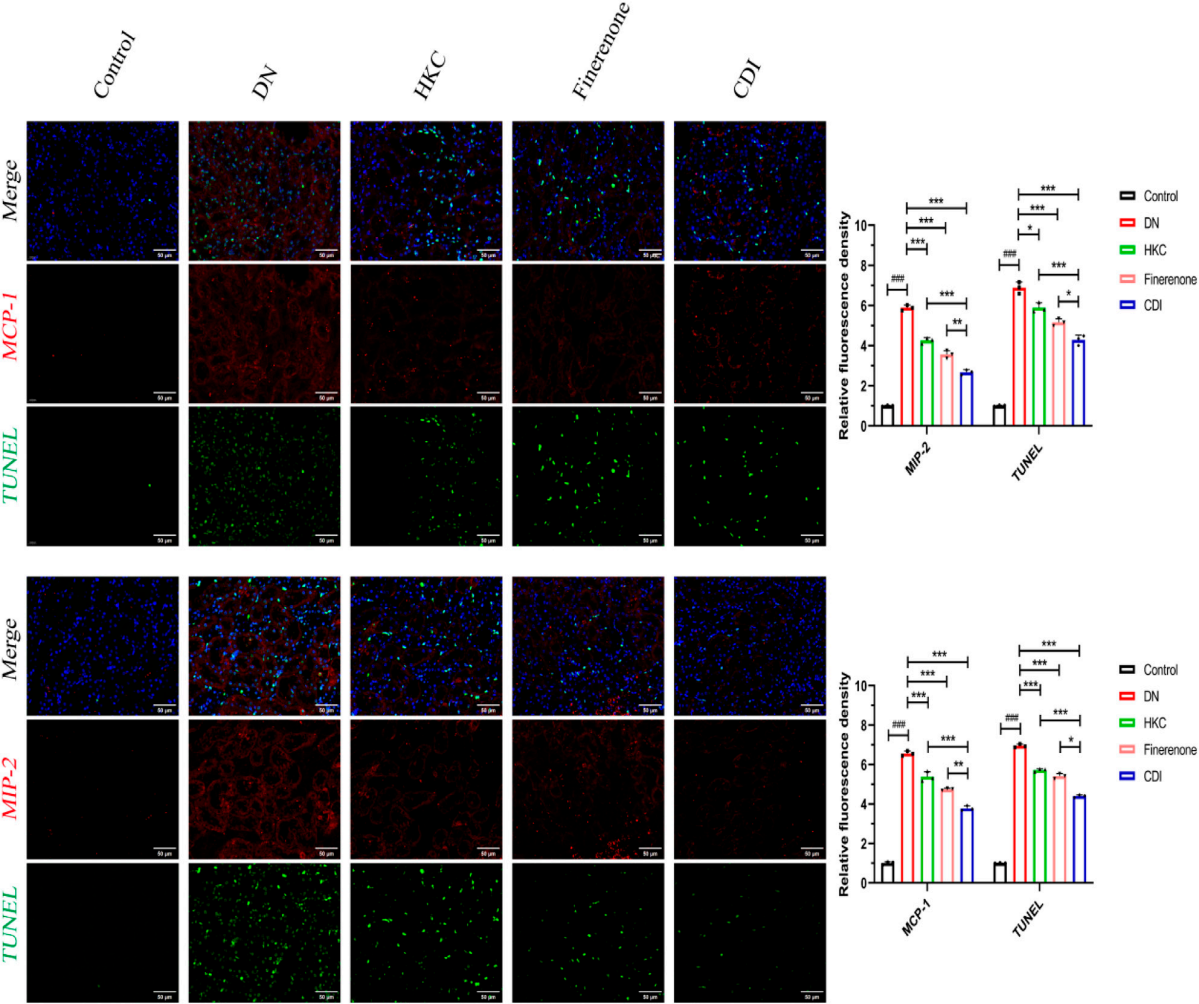
IF/TUNEL co-staining analysis of renal tubular inflammation and apoptosis in DN mice treated with combined drugs (n = 3 per group, scale bar 50 μm, ‾X ± SD), which was analyzed by one-way ANOVA. The CDI group showed reduced MCP-1/MIP-2 fluorescence and apoptosis rate vs. model (***p < 0.001) and vs. monotherapy (*p < 0.05) groups.

As shown in Figure 7, IF and TUNEL co-staining was used to analyze the relationship between the inflammatory proteins (MCP-1 and MIP-2) and apoptosis in the renal tubules of each group. Compared with that in the control group, the local renal tubular inflammation and nuclear apoptosis in the model group were significantly increased (p < 0.001). Compared with that in the model group, the effects of HKC, finerenone, and CDI on renal tubular inflammation and nuclear apoptosis were improved (p < 0.01), and the effects of the CDI group were better than those of the single-drug group (p < 0.05).

#### 4.2.6 Western blot

**FIGURE 8:**
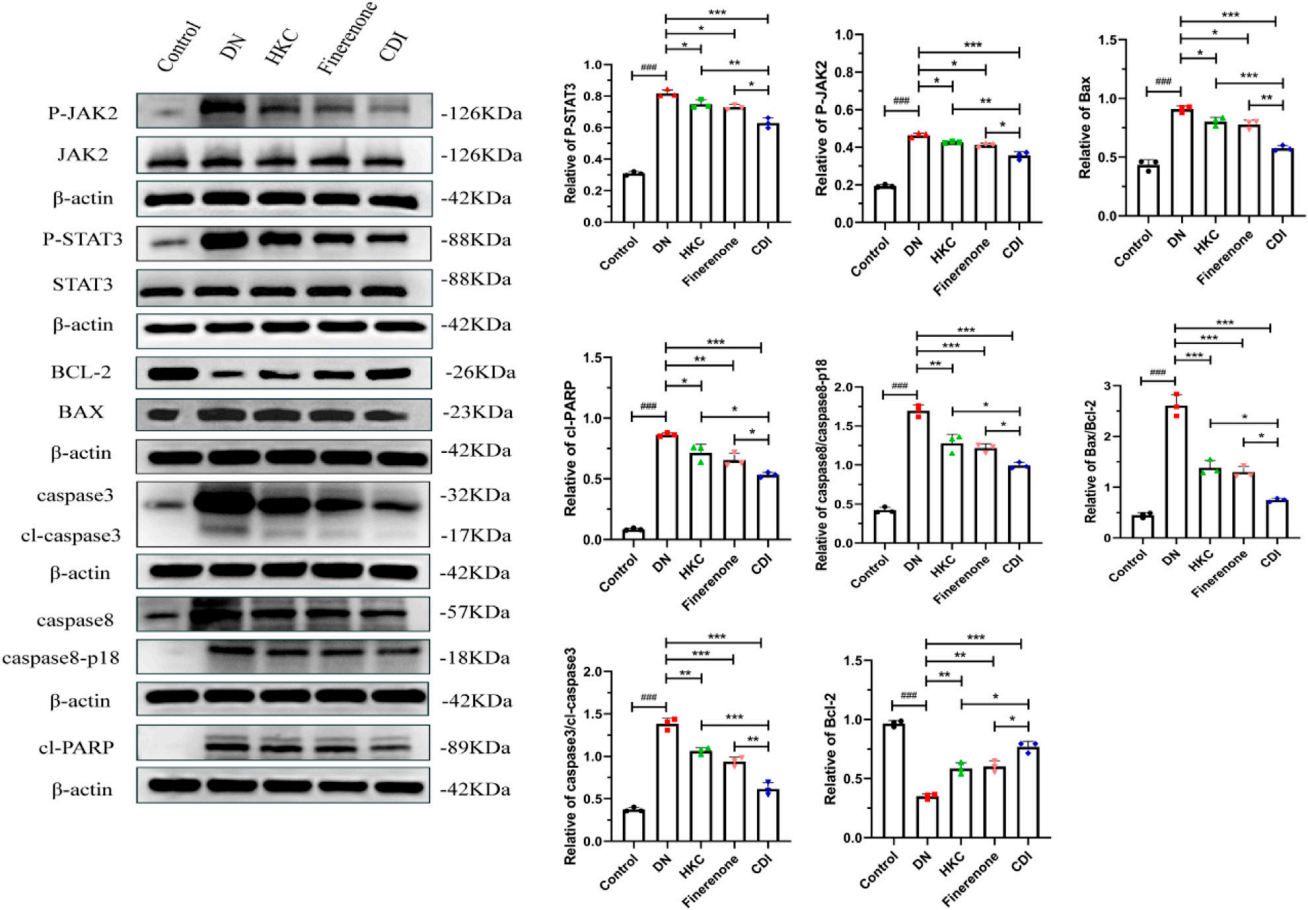
Western blot analysis of JAK2/STAT3 signaling pathway and apoptosis marker proteins in DN mice (n = 3 per group, ‾X ± SD): the CDI group suppressed p-JAK2, p-STAT3, Bax/Bcl2 ratio, and cleaved caspases vs. model (***p < 0.001) and vs. monotherapy (*p < 0.05) groups.

As depicted in Figure 8, the renal tissue of the model group exhibited elevated protein expression levels of p-JAK2, p-STAT3, Bax, caspase-3/cl-caspase-3, caspase-8/cl-caspase-8 (p18), and cl-PARP than the control group (p < 0.001), alongside an increased Bax/Bcl2 ratio (p < 0.001). Conversely, the Bcl2 protein expression was reduced (p < 0.001). Following drug intervention, the HKC, finerenone, and CDI groups showed decreased levels of p-JAK2, p-STAT3, Bax, caspase-3/cl-caspase-3, caspase-8/cl-caspase-8 (p18), and cl-PARP (p < 0.01), with an upregulation in the Bcl2 expression relative to the model group (p < 0.01). Notably, the CDI group demonstrated a more substantial reduction in these apoptotic markers and a higher Bcl2 expression level than the single-drug treatment groups (p < 0.05).

### 4.3 Cell experiment verification

#### 4.3.1 Determination of drug toxicity and combination index for Que and Fine

**FIGURE 9:**
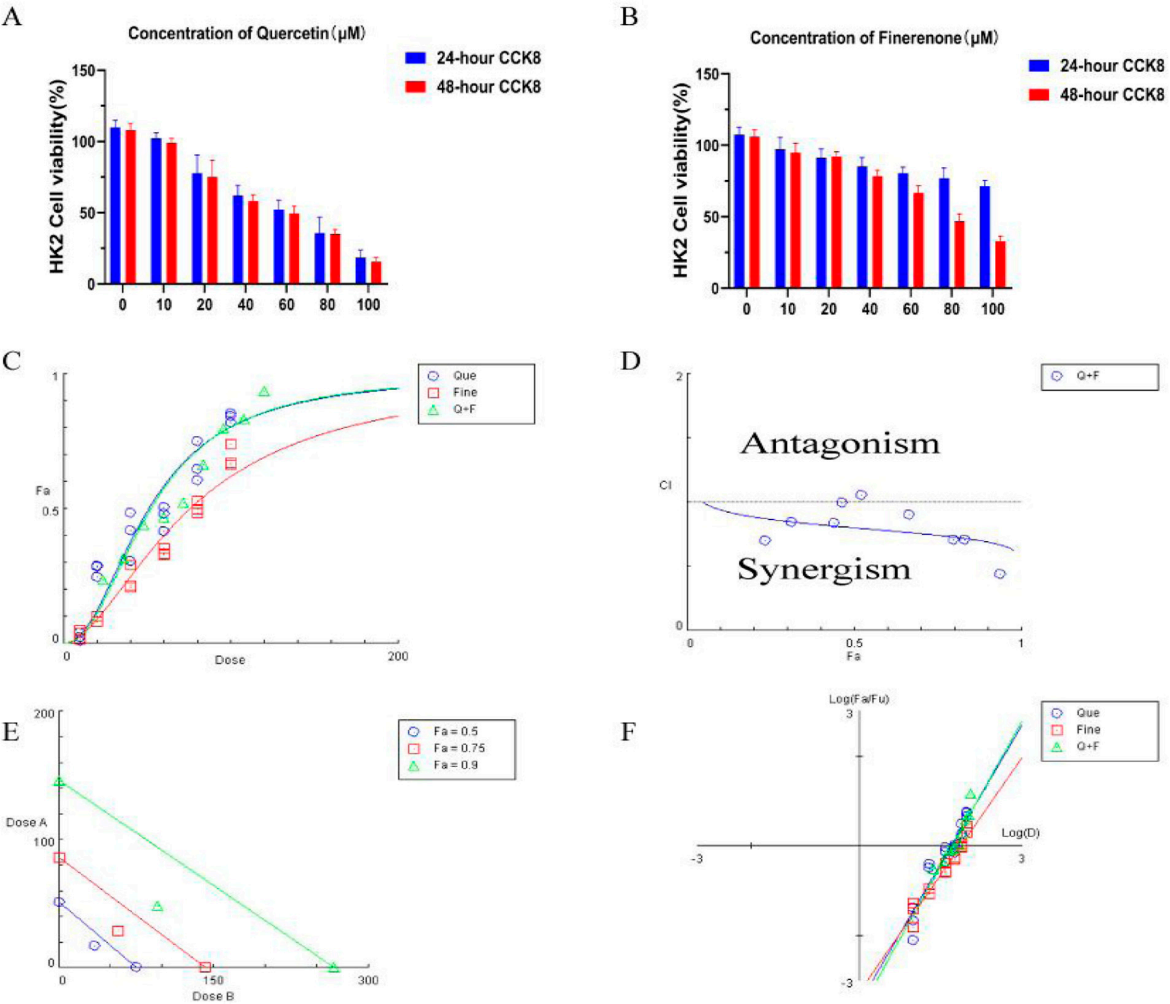
Drug toxicity of quercetin and finerenone on H-K2 cells: **(A,B)** (n = 6):CCK-8 assay was used to detect the drug toxicity and cell survival rate of HK-2 cells treated with quercetin and finerenone for 24 and 48 h, respectively. Synergistic effect of quercetin and finerenone in HK-2 cells. **(C)** Dose–effect curve, IC50 (quercetin = 50.75 μM, finerenone = 75.48 μM). **(D)** Chou–Talalay analysis. Combination index (CI = 0.80; Cl > 1, antagonistic effect; Cl = 1, additive effect; Cl < 1, synergistic effect) confirmed synergy. **(E)** Equivalent dose curve; the combination of quercetin and finerenone exhibited significant synergistic effects at inhibition rates of 50%, 75%, and 90%. **(F)** Median effect curve.

As shown in Figures 9A, B, CCK-8 was used to detect the drug toxicity of Que (0–100 μM) and Fine (0–100 μM) on HK-2 cells at 24 h and 48 h. In HK-2 cells treated with Que, compared with the 0 μM control group, the cell survival rate decreased continuously with the increase in the drug dose. At the concentration of 40 μM after 24 h and 48 h, the cell survival rate decreased significantly. To assess the inhibitory properties of the combined treatment, we validated the CCK8 data using CompuSyn software to obtain OD values after HK-2 cells were treated with Que and Fine in a 1:2 ratio for 24 h, and cell inhibition rates were calculated. In panel C, the dose–response curves of Que, Fine, and the combination drugs show the relationship between Fa and dose. Fig. F shows the median effect of each group, and then the semi-inhibitory concentration values of Que, Fine, and the combination drugs were deduced (Supplementary Table S7). In Figure 9D, based on Chou–Talalay method analysis, the combination index of Que and Fine showed that most of the nine combined data points showed a synergistic relationship (CI < 1), and HK-2 cells showed a strong synergistic behavior at 24 h of the combined drug intervention. The simulation at low Fa showed an obvious antagonistic effect. In panel E, allelic plots for 50%, 75%, and 90% inhibition are shown, and the Que + Fine data points on the diagonal, indicating synergy, are shown in the lower left. In this case, the half-inhibitory concentration, IC75, and IC90 indicate synergy.

#### 4.3.2 IF and apoptosis assays

**FIGURE 10:**
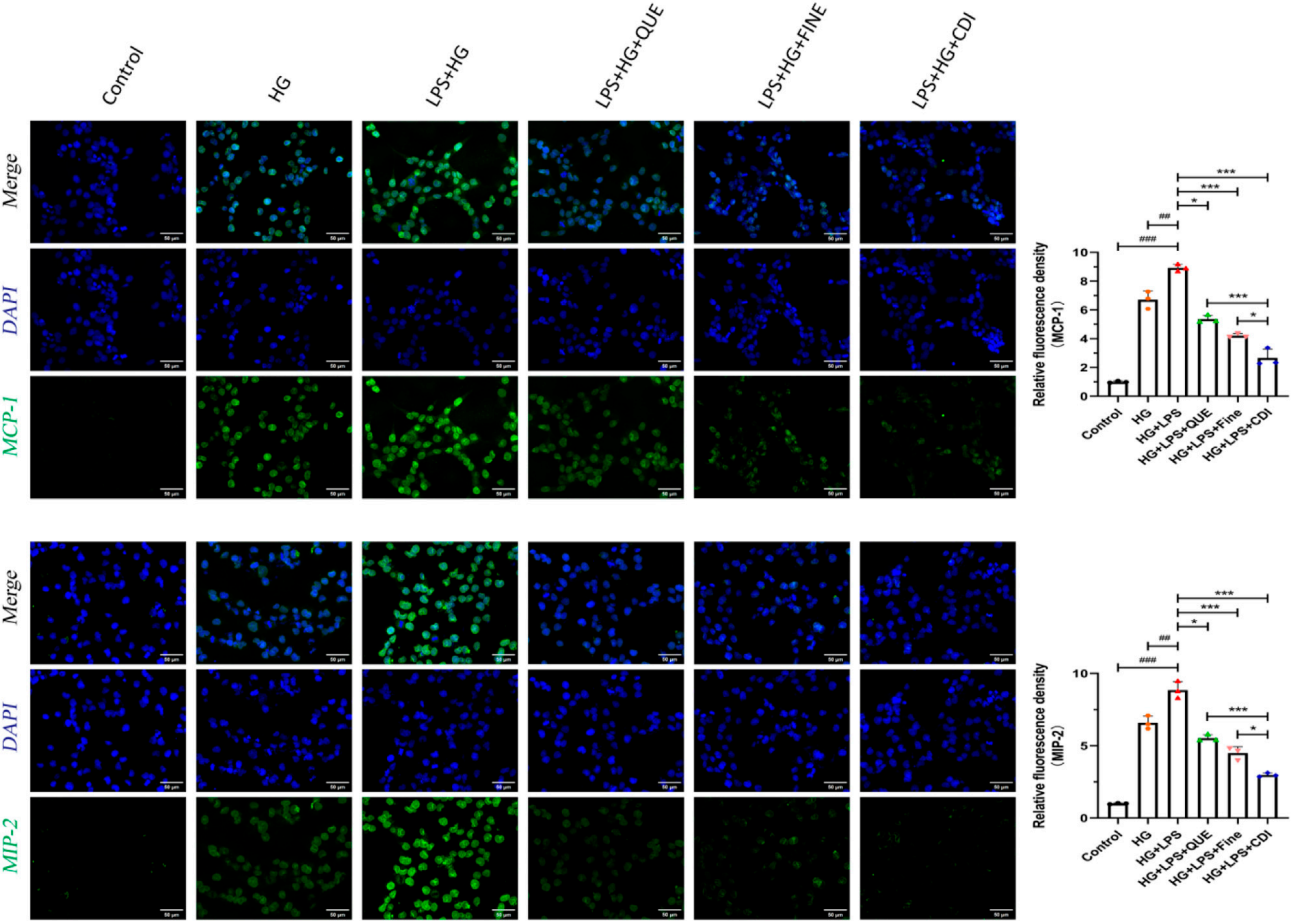
IF staining analysis of the anti-inflammatory effect of combined treatment on HK2 cells (n = 3 per group, scale bar 50 μm, ‾X ± SD): the CDI group showed reduced MCP-1/MIP-2 fluorescence by 70% vs. LPS + high glucose (***p < 0.001) and vs. monotherapy (*p < 0.05) groups.

**FIGURE 11:**
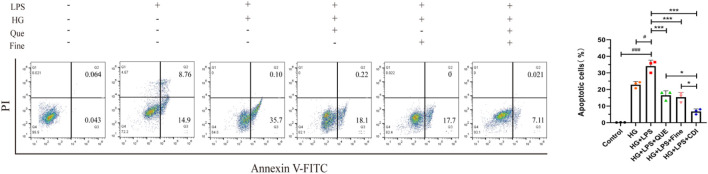
Flow cytometry analysis of the inhibitory effect of combined drugs on the apoptosis of HK-2 cells (n = 3 per group, ‾X ± SD), which was analyzed by one-way ANOVA. The CDI group showed reduced apoptosis rate vs. LPS + high glucose (***p < 0.001) and vs. monotherapy (*p < 0.05) groups.

According to the dose–effect curve and combined index value calculated by CompuSyn software, the intervention concentrations of Que and Fine were 15 μM and 30 μM, respectively. After 12 h of serum starvation before dosing, the intervention of each drug group was given according to the dosage of each group for 24 h. As shown in Figure 10, compared with the high-glucose group and the control group, the relative fluorescence quantitative values of inflammatory proteins MCP-1 and MIP-2 in HK-2 cells of the LPS + high glucose group were significantly increased (p < 0.01), indicating that the inflammation was successfully induced by LPS + high glucose. Compared with the LPS + high-glucose group, the relative fluorescence quantitative values of inflammatory proteins MCP-1 and MIP-2 in the Que, Fine, and CDI treatment groups decreased (p < 0.05), and those in the CDI treatment group decreased more significantly than in the single-drug treatment group (p < 0.05). As shown in Figure 11, compared with the high-glucose group and the control group, the percentage of apoptotic HK-2 cells in the LPS + high-glucose group was significantly increased (p < 0.05). Among them, the largest percentage of apoptosis was late apoptosis, indicating that the LPS + high glucose-induced apoptosis was successful. Compared with the LPS + high-glucose group, the percentage of apoptotic cells in the Que, Fine, and CDI intervention groups decreased (p < 0.001), and the CDI intervention group had a more significant reduction than the single-drug treatment group (p < 0.05).

#### 4.3.3 Western blot

**FIGURE 12:**
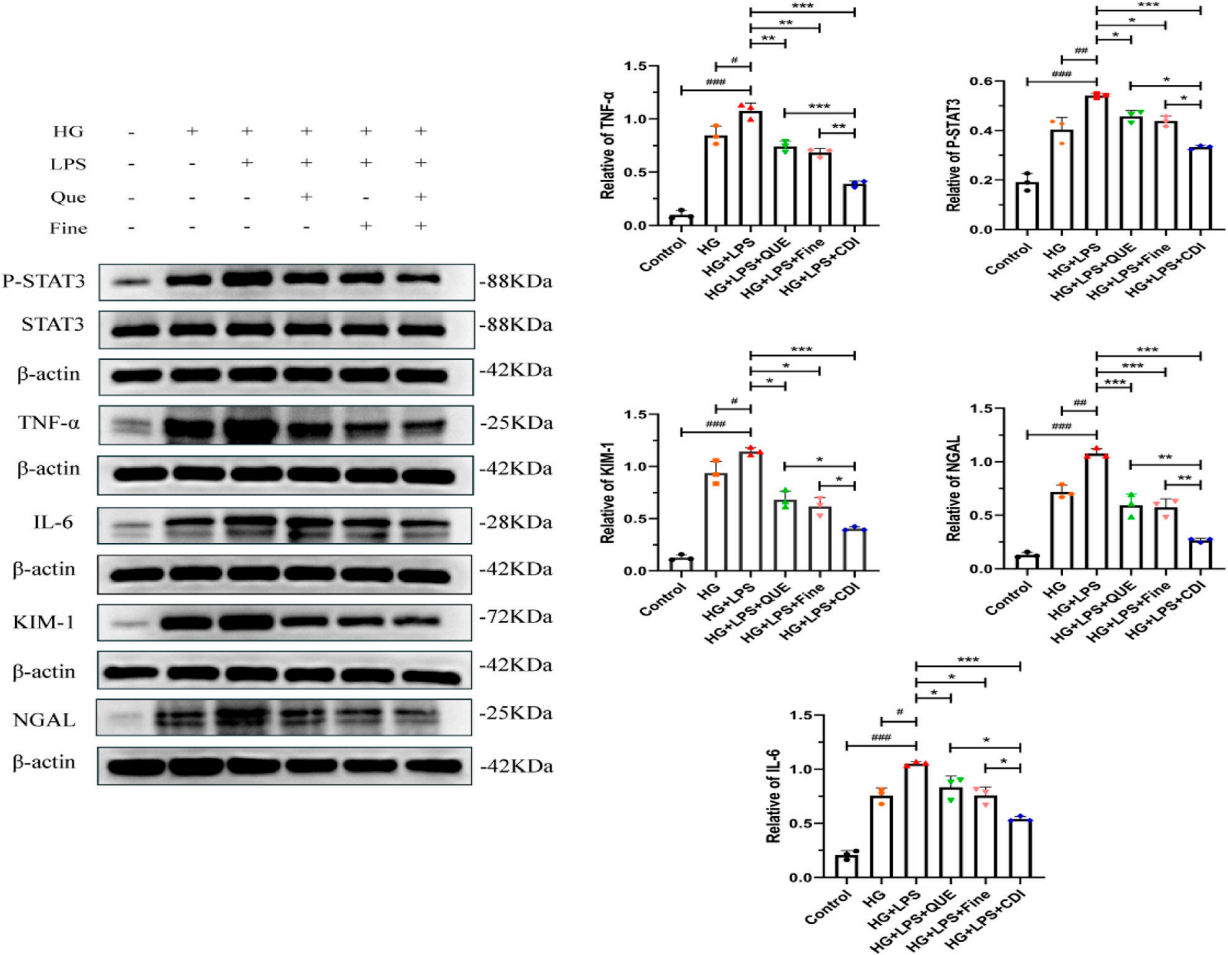
Western blotting analysis of the inhibition of inflammation, renal tubular injury markers, and STAT3 activation by the combination treatment before transfection in HK-2 cells (n = 3 per group, ‾X ± SD), which was analyzed by one-way ANOVA. The CDI group showed downregulated p-STAT3, IL-6, TNF-α, KIM-1, and NGAL vs. LPS + high glucose (***p < 0.001) and vs. monotherapy (*p < 0.05) groups.

The doses of Que and Fine were determined as previously described. As illustrated in Figure 12, the levels of P-STAT3, IL6, TNF-α, KIM-1, and NGAL proteins in HK-2 cells were markedly elevated in the LPS combined with high glucose group than in both the high-glucose group and the control group (p < 0.001). Furthermore, these protein levels were higher in the LPS + high glucose group than in the high-glucose group alone (p < 0.05). In contrast, the Que, Fine, and CDI treatment groups exhibited reduced expressions of P-STAT3, IL-6, TNF-α, KIM-1, and NGAL proteins than the LPS + high glucose group (p < 0.05). Notably, the CDI intervention group demonstrated a more pronounced decrease in these markers than the groups treated with either drug alone (p < 0.05).

#### 4.3.4 STAT3 was overexpressed by lentivirus transfection

As shown in Supplementary Figure S1, the optimal lentiviral infection titer (MOI = 10–100) for HK-2 cells was screened. Compared to MOI = 100 and 80, the viral usage and cell death were lower at MOI = 60. In comparison to MOI = 10–40, the transfection efficiency was optimal at MOI = 60. After 72 h of transfection, puromycin (5 μg/mL) was added for continuous screening, resulting in stable HK-2 cell lines overexpressing STAT3.

#### 4.3.5 Detection of apoptosis after overexpression of STAT3

As shown in Supplementary Figure S2, in the HK-2 cell line containing the stable overexpression of the STAT3 gene, the intervention was carried out according to the administration concentration and conditions mentioned above. Compared with stable HK-2 cell lines containing LV-NC and LV-STAT3, the percentage of apoptosis in stable HK-2 cell lines containing LV-NC and LV-STAT3 was significantly decreased after CDI intervention (p < 0.001). In addition, the percentage of apoptosis in each group of stable HK-2 cells containing LV-STAT3 was significantly higher than that in each group of stable HK-2 cells containing LV-NC (p < 0.001). These results demonstrate that CDI regulates cell apoptosis by regulating the phosphorylation of STAT3, a downstream target of the JAK2/STAT3 pathway (p < 0.001).

#### 4.3.6 Western blot after overexpression of STAT3

As shown in Supplementary Figure S3, different groups were treated under specific drug concentrations and experimental conditions. The results demonstrated that the protein expression levels of p-STAT3, IL-6, TNF-α, KIM-1, and NGAL in HK-2 cells with STAT3 overexpression were significantly higher than those in the overexpression control group (p < 0.05). This not only confirmed the stable overexpression of the STAT3 gene in HK-2 cells but also indicated that STAT3 overexpression exacerbated inflammation and injury in HK-2 cells. Following CDI intervention, the protein expression levels of p-STAT3, IL-6, TNF-α, KIM-1, and NGAL in both the overexpression control group and the STAT3-overexpressing group were markedly reduced (p < 0.001), suggesting that the combined drugs could modulate the JAK2/STAT3 signaling pathway to exert anti-inflammatory effects and mitigate tubular injury.

## 5 Limitations

This study has several limitations: (1). The pro-inflammatory/pro-apoptotic role of STAT3 was confirmed via overexpression, but its necessity remains incompletely validated due to the lack of genetic knockdown/knockout models. (2). Although quercetin was identified as a key active component, potential synergistic effects of other HKC flavonoids (e.g., myricetin and gossypetin) with finerenone were not explored, possibly underestimating the multi-target nature of traditional Chinese medicine. (3). Clinical translatability is limited by the absence of human data and long-term pharmacokinetic/toxicological assessments. (4). The sample size of the STZ-induced DN mouse model in this study was insufficient, and the adverse effects and safety following drug intervention were not adequately evaluated. (5). In the cell and animal experiments, only a single dose was used, and multiple doses were not used for comparison.

## 6 Discussion

Recent studies have established that early DN is characterized by distinct histopathological changes, including tubular cell hypertrophy, thickening of the tubular basement membrane (TBM), and interstitial inflammation mediated by monocyte infiltration (Pourghasem et al., 2015). Importantly, these TBM alterations represent primary pathological features rather than secondary consequences of glomerular hemodynamic changes, underscoring the central role of tubular injury in DN progression (Fioretto and Mauer, 2007).

Sustained hyperglycemia in DN promotes tubular inflammation, leading to apoptotic cell death and the release of tubular injury biomarkers, such as NGAL and KIM-1 (Satirapoj, 2018). Renal tubular epithelial cell apoptosis is a critical event in DN, which is driven by the synergistic activation of intrinsic (mitochondrial) and extrinsic (death receptor) pathways under hyperglycemic conditions. The intrinsic pathway involves oxidative stress-induced mitochondrial dysfunction, resulting in Bax/Bcl-2 imbalance, cytochrome C release, and caspase-9 activation. Meanwhile, the extrinsic pathway is initiated by inflammatory mediators such as TNF-α and Fas ligand (FasL), which engage death receptors tumor necrosis factor receptor 1/Fas ligand (TNFR1/Fas) and activated caspase-8. These pathways converge via BH3-interacting domain death agonist/truncated Bid (Bid/tBid)-mediated crosstalk, ultimately leading to caspase-3 activation and apoptotic execution. The resulting release of damage-associated molecular patterns (DAMPs) and proinflammatory cytokines (IL-1β and IL-6) further amplifies inflammation through NLRP3 inflammasome activation and JAK2/STAT3 signaling, creating a self-perpetuating cycle of inflammation and apoptosis. JAK2/STAT3 activation also upregulates chemokines (MCP-1 and MIP-2) and promotes neutrophil recruitment, which exacerbates renal injury and fibrosis (Riedl and Salvesen, 2007; Kesavardhana et al., 2020). (Supplementary Figure S4)

Combining TCM and Western medicine offers a promising strategy for DN treatment. This approach merges targeted drug therapy with TCM’s multi-target effects (Gong et al., 2024). Network pharmacology analysis identified quercetin, myricetin, and gossypetin as key active compounds in HKC. Topological analysis showed 11 shared targets between these flavonoids and finerenone. Molecular docking confirmed strong binding between quercetin–finerenone and JAK2/STAT3, a pathway central to DN via inflammation, fibrosis, autophagy, and apoptosis regulation (Xiong et al., 2024; Liu et al., 2023; Zhang et al., 2020; Zhong et al., 2024). Beyond JAK2/STAT3, other JAK/STAT members contribute to DN. STAT6 drives fibrosis through fibroblast activation and M2 macrophage polarization (Jiao et al., 2021a), while STAT1 worsens DN via p53/p21-mediated senescence (Jiao et al., 2021b). This signaling network also interacts with PI3K/AKT and TGF-β/Smad pathways, highlighting its broad regulatory role. HKC exerts renal protection beyond JAK2/STAT3 inhibition. It activates the kelch-like ECH-associated protein 1/nuclear factor erythroid 2-related factor 2/heme oxygenase 1(Keap1/Nrf2/HO-1) axis, reducing oxidative stress in diabetic kidneys (Su et al., 2025). Metabolomic studies show that HKC suppresses the pro-fibrotic 5-HT/5-HT2AR pathway in tryptophan metabolism. Additionally, HKC flavonoids may regulate phosphatase and tensin homolog (PTEN) and cyclic GMP-AMP synthase/stimulator of interferon gene (cGAS/STING) pathways, which control inflammation and fibrosis (An et al., 2022; Jiao et al., 2025). PTEN loss disrupts Nrf2 antioxidant responses, while cGAS/STING overactivation worsens DN inflammation. Thus, HKC may preserve renal function by balancing PTEN and STING activity. Quercetin exemplifies HKC’s multi-target action, inhibiting JAK2/STAT3 expression while activating that of PI3K/AKT. This dual modulation underscores the benefit of combined therapies. Further research should clarify how these pathways interact to enhance treatment efficacy.

DN progression is marked by tubular inflammation, apoptosis, and fibrosis, collectively leading to decreased renal function (Rohm et al., 2022; Wada and Makino, 2016; Shi et al., 2018). Our results demonstrate that HKC–finerenone combination therapy significantly reduced proteinuria and improved renal function in DN mice, correlating with attenuated tubular vacuolization, collagen deposition, and inflammatory infiltration. The combination synergistically suppressed apoptosis and inflammation by inhibiting JAK2/STAT3 activation, thereby reducing IL-6/TNF-α production, caspase-8-mediated apoptosis, and mitochondrial Bax translocation. This dual inhibition was further validated by the Chou–Talalay synergy index in HK-2 cells. STAT3 overexpression exacerbated inflammation and apoptosis, which were reversed by combined treatment, confirming the pathway’s central role. The HKC–finerenone interaction requires evaluation for clinical translation. Finerenone is mainly metabolized by cytochrome P450 family 3 subfamily A member 4 (CYP3A4) (Heinig and Eissing, 2023), and HKC flavonoids (e.g., quercetin) show moderate CYP3A4 inhibition *in vitro* (Ai et al., 2023; Wang et al., 2025). However, quercetin’s low bioavailability and rapid metabolism likely minimize this effect. Mechanistically, finerenone blocks aldosterone-induced fibrosis, while HKC reduces oxidative stress and STAT3-driven inflammation. Our isobolographic analysis confirms the synergy (Cl:0.42–0.68). Clinically, both drugs have proven safety (finerenone is FDA-approved for diabetic kidney disease; HKC is listed in the Chinese Pharmacopoeia with renal safety data). Future phase-I dose-escalation studies should refine dosing, especially with CYP3A4 inhibitors.

In summary, our study integrates network pharmacology and experimental validation to elucidate the synergistic mechanism of HKC–finerenone in DN. The combination targets JAK2/STAT3 to disrupt the inflammation–apoptosis cycle, offering a novel tubular-centric therapeutic strategy. Future studies should explore multi-component interactions and long-term safety to facilitate clinical translation.

## Data Availability

The datasets presented in this study can be found in online repositories. The names of the repository/repositories and accession number(s) can be found in the article/Supplementary Material.
