# Supplementary material for: Huangkui capsule combined with finerenone attenuates diabetic nephropathy by regulating the JAK2/STAT3 signaling pathway based on network pharmacology, molecular docking, and experimental verification
Source: Front Pharmacol. 2025 Aug 4;16:1625286. doi: 10.3389/fphar.2025.1625286 (PMC12358392; doi:10.3389/fphar.2025.1625286)
Supplement: Supplementary file 1 [file DataSheet1.pdf]

Supplementary material

Supplementary material associated with this article can be found, in the online version, at there：

Table 1. Core components of DN treated by HKC and Finerenone granules

MOL ID	Compound	Degree	From	OB	DL	Abbreviations
MOL000098	Quercetin	101	PMF	46.3	0.28	QUE
MOL002008	Myricetin	101	PMF	13.75	0.31	MYR
MOL004020	Gossypetin	101	PMF	35	0.31	GOS
MOL105047	Finerenone	100	MRA	/	/	FINE
MOL001787	Adenosin	72	OA	15.98	0.18	ADO
MOL013374	Dihydromyricetin	71	PMF	23.48	0.31	DMY
MOL004718	α-spinasterol	58	SDRR	42.98	0.76	α-SPI
MOL002687	Guanosine	49	OA	21.43	0.21	GR
MOL001987	β-sitostero	44	SDRR	33.94	0.7	β-SIT
MOL000449	Stigmasterol	43	SDRR	43.83	0.76	STG
MOL012378	4',5,7,8-Tetramethoxyflavone	21	PMF	23.45	0.36	TMF
MOL000875	Cedrol	12	OA	16.23	0.12	CED
MOL001955	Chlorogenic acid	7	OA	11.93	0.33	CGA
MOL001398	Methyl linolenate	7	OA	46.15	0.17	MLN
MOL000357	Sitogluside	3	SDRR	20.63	0.62	SITO
MOL005521	Phytane	3	OA	13.86	0.11	PHY

Notes: Sixteen bioactive compounds (15 from HKC and finerenone) were identified through network pharmacology screening. Degree values reflect the number of target interactions, with higher values indicating broader pharmacological effects. Compoun ds are classified by origin: PMF (plant-derived flavonoids), MRA (mineralocorticoid receptor antagonist), SDRR (steroid derivat ives), and OA (other active compounds). OB≥10% and DL≥0.1 were applied to ensure pharmacologically relevant candidates. T he top three HKC components (quercetin, myricetin, gossypetin) showed the highest degree values (101), suggesting their pivot al roles in DN treatment.

Table 2. Calculation of ligand-protein binding free energy by MM-PBSA (kcal/mol)

Complex	ΔEvdW	ΔGelec	ΔGGB	ΔGSA	ΔGbind
STAT3-Quercetin	-134.465	-69.532	156.642	-18.111	-65.465
STAT3-Finerenone	-107.668	-56.835	141.709	-17.622	-40.416
JAK2-Finerenone	-115.330	-44.733	147.895	-18.580	-30.748
JAK2-Quercetin	-102.711	-11.215	76.355	-15.650	-53.221

Notes：Molecular dynamics simulations were performed to calculate binding free energies (ΔGbind) between core targets (STA T3/JAK2) and ligands (quercetin/finerenone) using MM-PBSA. Energy components include: ΔEvdW (van der Waals interaction s), ΔGelec (electrostatic energy), ΔGGB (polar solvation energy), and ΔGSA (non-polar solvation energy). The STAT3-querce tin complex exhibited the strongest binding affinity (ΔGbind = -65.465 kcal/mol), consistent with molecular docking results. N egative ΔGbind values indicate spontaneous binding, with lower values reflecting greater stability.

Table 3. Urine protein was quantified 24 h in each group（mg/L, $\bar{X}$±SD，*n*=8）

Group	Week 0	Week 2	Week 4	Week 6	Week 8
Control	84.62±8.76	82.05±6.83	89.10±9.58	83.71±8.13	82.08±6.49
DN	117.20±16.40	177.27±8.55$^{\#\#\#}$	271.90±14.03$^{\#\#\#}$	770.75±57.83$^{\#\#\#}$	1046.48±72.84$^{\#\#\#}$
HKC	124.34±17.12	173.93±12.52	226.43±6.17*	385.18±57.62***	587.62±49.28***
Finerenone	117.09±22.75	160.82±8.41	213.73±3.87**	208.78±43.91$^{***/*}$	421.75±48.83$^{***/**}$
CDI	115.36±19.68	157.20±7.35	193.83±7.16***	257.32±26.69***	358.54±21.21***

Notes：24-hour urinary protein levels were measured weekly in DN mice treated with HKC, finerenone, or combination therapy (CDI). Data were analyzed by two-way ANOVA with post-hoc tests. Symbols denote significance: *p<0.05 vs. model group; *p<0.05 vs. CDI group; $^{\#}$p<0.05 vs. control. The CDI group showed maximal proteinuria reduction (358.54±21.21 mg/L at we ek 8 vs. 1046.48±72.84 mg/L in model group, ***p<0.001), (CDI group at week 8 vs. Monotherapyl group, **p<0.01), demo nstrating synergistic renoprotection.

Table 4. Changes in body weight with weeks of age in each group of mice（g, $\bar{X}$±SD, *n*=8）

Group	Week 0	Week 2	Week 4	Week 6	Week 8
Control	23.42±1.00	25.68±0.86	27.36±0.73	28.17±1.04	30.63±1.56
DN	32.01±2.00	30.72±1.59	28.79±0.95	27.73±1.02	23.86±1.00
HKC	31.72±1.90	30.27±1.30	28.75±1.11	27.09±0.76	27.12±0.82
Finerenone	31.12±1.79	30.13±1.20	28.68±0.94	29.05±0.51	28.51±0.94
CDI	31.37±1.99	29.96±1.64	28.50±0.60	29.19±0.85	27.86±0.51

Notes：STZ successfully induced the establishment of a diabetic mouse model, with changes in body weight and weeks observ ed across each group of mice.

Table 5. Fasting blood glucose as a function of week age in each group of mice（mmol/L, $\bar{x}\pm SD$, n=8）

Group	Week 0	Week 2	Week 4	Week 6	Week 8
Control	5.58±0.75	5.40±0.70	6.00±0.90	5.25±0.79	5.58±0.87
DN	18.04±0.74	19.56±0.83	21.04±1.19	21.60±1.61	22.63±1.05
HKC	17.18±0.68	18.61±1.02	20.79±1.58	20.16±0.90	20.30±1.18
Finerenone	17.68±0.82	18.91±0.88	20.63±1.25	20.32±0.97	20.70±1.03
CDI	17.61±0.65	19.01±1.26	19.86±1.19	19.45±0.81	19.81±1.39

Notes：The diabetic mouse model was successfully established using STZ induction. The changes in blood glucose levels acros s all groups were monitored over the course of several weeks.

Table 6. Sequences of the primers for real-time PCR

Mouse Gene	Forward	Reverse
JAK2	5'-ACAGACAAGTGGAGCTTCGG-3'	5'-CAGGCCTGAAATCTGGCTCA-3'
STAT3	5'-GCCAAATGCTTGGGCATCAA-3'	5'-AGGTTCCAATTGGCGGCTTA-3'
Bax	5'-ATGAAGACAGGGGCCTTTTTG-3'	5'-AATTCGCCGGAGACACTCG-3'
Bcl-2	5'-ATGCCTTTGTGGAACTATATGGC-3'	5'-GGTATGCACCCAGAGTGATGC-3'
Caspase3	5'-TGTCGATGCACCTGGACAAC-3'	5'-TGCTTGTCGGCAGTGTCAGAG-3'
Caspase8	5'-ACATGGCACCTTGCTGACTT-3'	5'-GGCTCAGAGTGATGCCATTT-3'
PARP	5'-AGCTGACCTTGCTGACTGTTG-3'	5'-GGCTCATCACCTTCACCAGA-3'
β-actin	5'-TGTTACCAACTGGGACGATATGG-3'	5'-GATCCACATCTGCTGGAAGGTGG-3'

Notes：Primer sequences were designed using NCBI Primer-BLAST and validated by melt curve analysis. β-actin served as the endogenous control for normalization. Gene-specific primers targeted apoptosis-related markers (Bax, Bcl-2, caspases) and JAK 2/STAT3 pathway components. All primers showed 90-110% amplification efficiency in validation experiments.

Table 7. IC50 and CI values for Que (0–100 μM) and Fine (0–100 mM) single dose or in combination in HK2 cells for 24 h

Dose Strategy	IC50/Dm values	Cl values for HK2
Que（μM）	50.7484	-
Fine（μM）	75.4757	-
Que+Fine	51.9683	0.80037

Notes：Dose-response curves were generated using CCK-8 assays after 24 h treatment. IC50 values (half-maximal inhibitory co ncentration) were calculated by CompuSyn software. Combination index (CI) was determined via Chou-Talalay method: CI<1 i ndicates synergy; CI=1 additive effect; CI>1 antagonism. Quercetin-finerenone (1:2 ratio) showed synergistic inhibition of HK2 apoptosis (CI=0.80), with IC50 values of 50.75 μM (quercetin) and 75.48 μM (finerenone).

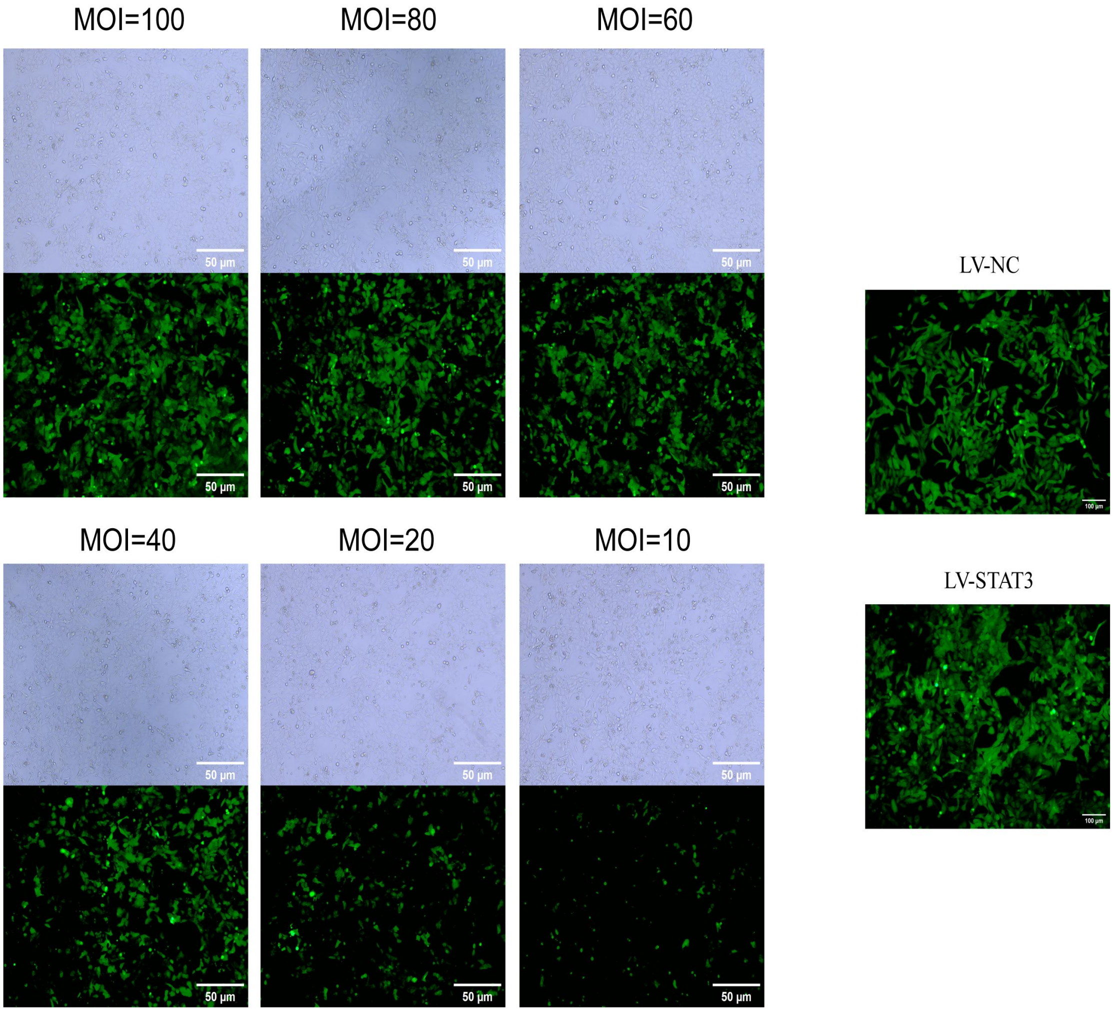

Figure 1. Overexpression of STAT3 in HK2 cells by lentiviral transfection (Scale bar 50μm) : Lentiviral transfection:White field and fluorescence control plots of cells. Optimal MOI=60 achieved 85% transfection efficiency without cytotoxicity.

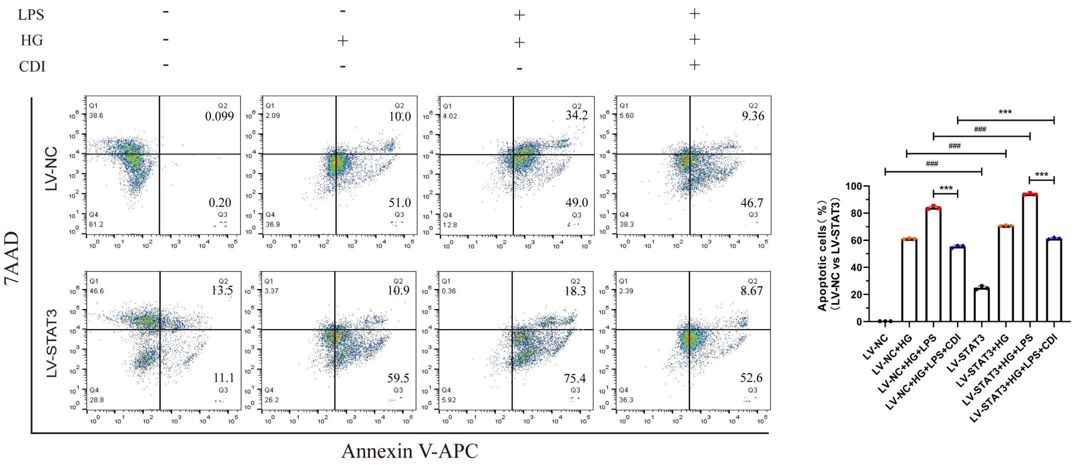

Figure 2. Flow cytometry analysis showed that STAT3 overexpression aggravated HK2 cell apoptosis induced by LPS and high glucose, which was reversed by combined treatment (n=3 per group, $\bar{X} \pm SD$): Analyzed by one-way ANOVA. CDI group significantly reduced apoptosis in STAT3-overexpressing cells vs. LPS+High glucose+LV-STAT3 group (*p<0.001). Additionally, the CDI group showed lower apoptosis rates vs. LPS+High glucose+LV-NC group (p<0.01), demonstrating the synergistic protective effect of the combination therapy.

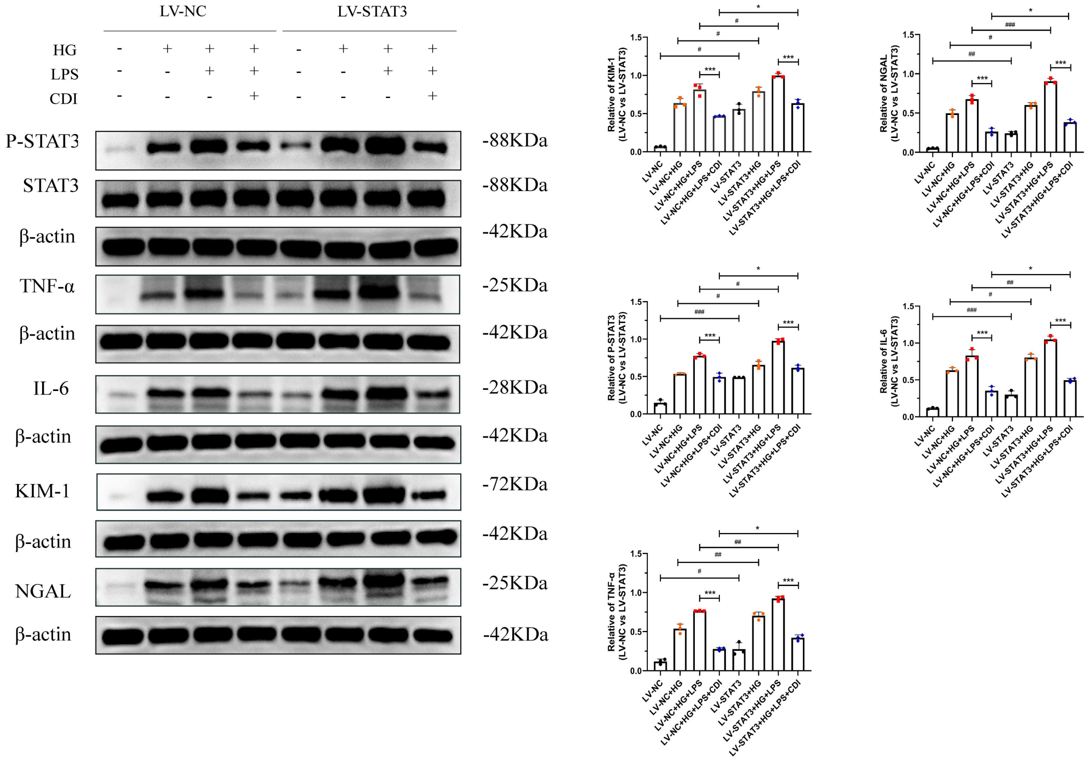

Figure 3. Western blot analysis showed that overexpression of STAT3 aggravated renal tubular injury in HK2 cells induced by LPS plus high glucose and was reversed by the combination drug (n=3 per group, $\bar{X} \pm SD$): Analyzed by one-way ANOVA. CDI group suppressed STAT3 overexpression-induced inflammation and injury markers (p-STAT3, IL-6, TNF-α, KI

M-1, NGAL) vs. LPS+High glucose+LV-STAT3 group (*p<0.001). Furthermore, the CDI group exhibited superior efficacy vs. LPS+High glucose+LV-NC group (p<0.01), confirming the therapeutic advantage of the combined intervention.

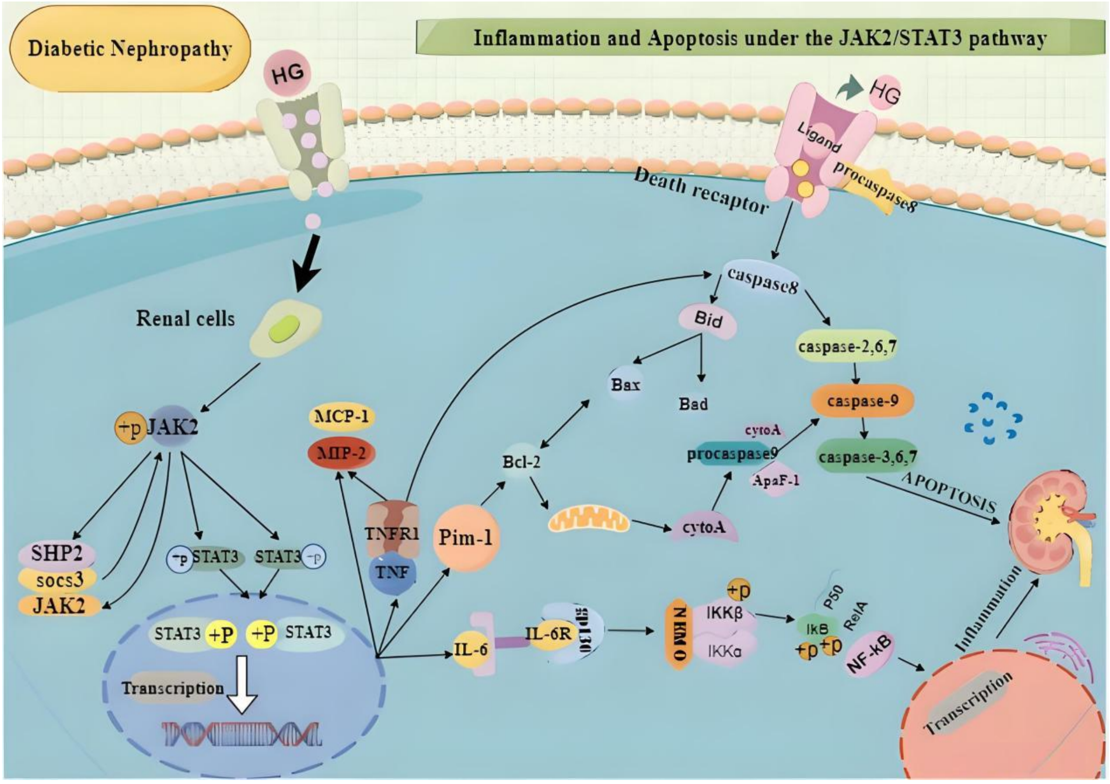

Figure 4. Schematic illustration of the JAK2/STAT3 signaling pathway in DN: Under hyperglycemic conditions , persistent JAK2/STAT3 activation promotes: (1) Transcriptional upregulation of pro-inflammatory cytokines (IL-6, TNF-α) and chemokine s (MCP-1, MIP-2) through STAT3 nuclear translocation; (2) Mitochondrial apoptosis via increased Bax/Bcl-2 ratio and caspase-3/8 activation; (3) Tubular injury manifested by elevated NGAL and KIM-1 expression.
